# Why do lactic acid bacteria thrive in chain elongation microbiomes?

**DOI:** 10.3389/fbioe.2023.1291007

**Published:** 2024-01-11

**Authors:** Barbara Ulčar, Alberte Regueira, Maja Podojsteršek, Nico Boon, Ramon Ganigué

**Affiliations:** ^1^ Center for Microbial Ecology and Technology (CMET), Department of Biotechnology, Ghent University, Gent, Belgium; ^2^ Center for Advanced Process Technology for Urban Resource Recovery (CAPTURE), Gent, Belgium; ^3^ Department of Chemical Engineering, Universidade de Santiago de Compostela, Santiago de Compostela, Spain

**Keywords:** chain-elongating bacteria, granular sludge, caproic acid, growth parameters, product inhibition, R/K strategies

## Abstract

Efficient waste management is necessary to transition towards a more sustainable society. An emerging trend is to use mixed culture biotechnology to produce chemicals from organic waste. Insights into the metabolic interactions between community members and their growth characterization are needed to mediate knowledge-driven bioprocess development and optimization. Here, a granular sludge bioprocess for the production of caproic acid through sugar-based chain elongation metabolism was established. Lactic acid and chain-elongating bacteria were identified as the two main functional guilds in the granular community. The growth features of the main community representatives (isolate *Limosilactobacillus musocae* G03 for lactic acid bacteria and type strain *Caproiciproducens lactatifermentans* for chain-elongating bacteria) were characterized. The measured growth rates of lactic acid bacteria (0.051 ± 0.005 h^−1^) were two times higher than those of chain-elongating bacteria (0.026 ± 0.004 h^−1^), while the biomass yields of lactic acid bacteria (0.120 ± 0.005 g biomass/g glucose) were two times lower than that of chain-elongating bacteria (0.239 ± 0.007 g biomass/g glucose). This points towards differential growth strategies, with lactic acid bacteria resembling that of a r-strategist and chain-elongating bacteria resembling that of a K-strategist. Furthermore, the half-saturation constant of glucose for *L. mucosae* was determined to be 0.35 ± 0.05 g/L of glucose. A linear trend of caproic acid inhibition on the growth of *L. mucosae* was observed, and the growth inhibitory caproic acid concentration was predicted to be 13.6 ± 0.5 g/L, which is the highest reported so far. The pre-adjustment of *L. mucosae* to 4 g/L of caproic acid did not improve the overall resistance to it, but did restore the growth rates at low caproic acid concentrations (1–4 g/L) to the baseline values (i.e., growth rate at 0 g/L of caproic acid). High resistance to caproic acid enables lactic acid bacteria to persist and thrive in the systems intended for caproic acid production. Here, insights into the growth of two main functional guilds of sugar-based chain elongation systems are provided which allows for a better understanding of their interactions and promotes future bioprocess design and optimization.

## 1 Introduction

The transition towards a more sustainable society requires efficient waste management. Besides lowering waste production, the conversion of waste into useful products is essential to enable resource circularity (Arancon et al., 2013; Iragavarapu et al., 2023). Mixed microbial communities can be used in open-culture bioprocess technologies for the production of chemicals, materials, and fuels from organic waste. Mixed communities can efficiently convert complex organic waste into products of interest (Colombo et al., 2017; Nzeteu et al., 2018; Bühlmann et al., 2022; Greses et al., 2022). Recent waste valorization technologies have shifted from biogas production towards products with a higher market value. In this context, the carboxylate platform has been proposed, where the last step of the anaerobic digestion process is inhibited and the accumulation of short-chain carboxylic acids (SCCA) (e.g., acetate, lactate) and other small molecules (e.g., ethanol) is targeted. The SCCA are an interesting product with various applications, however, the downstream processing steps of SCCA extraction are currently economically unfeasible due to their high water solubility (Moscoviz et al., 2018). As an alternative, the production of medium-chain carboxylic acids (MCCA), with carbon chains between 6–12 carbon atoms has been proposed. These have a higher market value and lower water solubility which makes their extraction less energy-intensive (Agler et al., 2011). Of specific interest is caproic acid (CA), a monocarboxylic acid with six carbon atoms, which is considered a platform chemical and is commercially used in the production of fragrances, pharmaceuticals, lubricants, rubbers, and dyes. Furthermore, CA can be used directly as an animal feed or as a food preservative (Cavalcante et al., 2017) due to its inhibitory effect on the growth of microorganisms (Palmqvist and Agerdal, 2000; Royce et al., 2013). On the other hand, the same inhibitory effects must be taken into account when considering the bio-production of CA. Characterization of microbial community members for their tolerance to CA is required to support bioprocess optimization.

The MCCA are produced by microorganisms in the so-called chain elongation (CE) metabolism through the cyclic reverse β-oxidation pathway (Agler et al., 2011; Angenent et al., 2016). In this pathway, an electron donor (e.g., glucose, lactate, ethanol) is used to elongate the carbon chain of an electron acceptor (e.g., acetate, propionate, butyrate) two carbon units per cycle. For instance, butyrate is produced using acetate as an electron acceptor, caproate is produced from butyrate, valerate from propionate, etc. Most microorganisms can only use one type of electron donor (Thauer et al., 1968; Kim et al., 2015; Flaiz et al., 2020; Esquivel-Elizondo et al., 2021; Gu et al., 2021; Kang et al., 2022), although recently new isolates have been identified that are able to grow on multiple (Wang et al., 2022b; Liu et al., 2022). For instance, the bacterium *Caproiciproducens lactatifermentans* can use glucose and/or lactate as an electron donor, showing the flexibility of metabolism to adjust to the available substrate (Wang et al., 2022b). In mixed culture bioprocesses intended to produce CA, other microorganisms are present besides chain-elongating bacteria (CEB); their usual companions are lactic acid bacteria (LAB) (Candry and Ganigué, 2021). The LAB ferment carbohydrate molecules to solely lactate (homofermentative metabolism) or to a mixture of lactate, ethanol, acetate, and some other small organic molecules (heterofermentative metabolism) (Spector, 2009). Although LAB can be difficult to enrich in a continuous bioprocess due to their disability to synthesize all the necessary amino acids and vitamins (Teusink and Molenaar, 2017; Rombouts et al., 2020), they are commonly present in continuous CE bioprocesses (Candry and Ganigué, 2021). It is not clear why LAB are persistently found in the CE systems, how they endure high CA concentrations in the system, and how they interact with other community members, especially with the CEB.

Considering the metabolism of LAB and CEB, their co-existence in a CE community enables two types of metabolic interactions. They may compete for the same substrate (i.e., sugars), or CEB may grow on lactate (and/or ethanol), the metabolic product(s) of LAB. Understanding the carbon flow between LAB and CEB is crucial for future knowledge-driven bioprocess optimization (Candry and Ganigué, 2021). The mere existence of LAB in CE systems indicates that this guild is better or, at least, as good as CEB at consuming sugars. Many studies suggest that in a system where sugars or complex medium are provided, the CEB cross-feed on the lactate produced by LAB (Scarborough et al., 2018b; Nzeteu et al., 2018; Carvajal-Arroyo et al., 2019; Lambrecht et al., 2019; De Groof et al., 2020; Mariën et al., 2022a). However, no research has directly investigated the carbon flows in these systems. Not only LAB but also some CEB are (theoretically) capable of lactate and acetate production from sugars (Esquivel-Elizondo et al., 2021), which can both later serve as substrates for the production of caproate. Due to the various possible combinations of reactions, the labelled substrate approach is difficult to apply to address the question of metabolic interactions between LAB and CEB. Alternatively, another, more ecological approach can be used to predict the substrate flow in mixed communities based on the growth parameters of individual organisms (i.e., growth rate, biomass yields, and substrate affinity). The r/K-selection theory can be applied to describe the behavior of organisms at various substrate concentrations in the environment, which can further be used to adjust the bioprocess operational parameters and steer the functioning of the microbial community (Andrews and Harris, 1986; Yin et al., 2022).

Optimization of mixed-culture bioprocesses can be achieved using two distinct approaches: top-down and bottom-up (Lawson et al., 2019). The top-down approach is more traditionally used in the field of environmental engineering, where various physicochemical parameters are tested and modeled for their impact on the bioprocess performance. The bottom-up approach revolves around the study and characterization of the individual members of the microbial community. The knowledge gathered this way can then be used to better understand the functioning of the mixed-culture system. This approach has been recognized as equally important to the bottom-down approach and as one of the key strategies towards knowledge-driven bioprocess optimization (Lawson et al., 2019; Candry and Ganigué, 2021). In this work, the growth of representatives of LAB and CEB from a sugar-based CE system was characterized to investigate their ecological life mode. Furthermore, the growth of LAB was characterized in the presence of CA to determine the inhibitory effect of CA. Ultimately, this knowledge allows to infer the metabolic interactions between the selected guilds and the implications for (granular) bioprocesses design and operation.

## 2 Materials and methods

### 2.1 Community representatives and medium design

#### 2.1.1 Reactor operation

A bioprocess for the production of caproic acid using granular sludge was set up to serve as the source for the isolation of community members. A glass up-flow expanded granular sludge bed (EGSB) reactor similar to that described in Carvajal-Arroyo et al. (2019) with a working volume of 2.4 L was operated at a hydraulic retention time of 1 day, at a pH of 5.5, and temperature of 34°C. The reactor was inoculated with the granular sludge from the reactor described in Mariën et al. (2022a), and was fed with a synthetic medium (Supplementary Table S1) containing glucose as a carbon source and including complex nitrogen sources (both tryptone and yeast extract) (Mariën et al., 2022a). The reactor was operated for 45 days, granular biomass was present and caproic acid was produced during the whole period of operation (Supplementary Figure S1).

#### 2.1.2 *Limosilactobacillus mucosae* G03 isolation and bacterial growth

A strain of lactic acid bacteria was isolated from the aforementioned reactor using the isolation by plating technique. Medium plates were prepared by adding 15 g/L of agar to the reactor medium (Supplementary Table S1), which contained 5 g/L of glucose instead of 20 g/L. Granular biomass was freshly sampled from the bioreactor, stored for the community structure analysis (Section 2.6.1), and used to isolate community members. A sample of 0.5 mL was anaerobically (in an N_2_ serum bottle) suspended in 10 mL of filter-sterilized (0.22 μm filter) reactor effluent. After this point, all the steps were performed inside an anaerobic (10% CO_2_, 90% N_2_) chamber (GP-Campus, Jacomex, TCPS NV, Rotselaar, Belgium). The sample was further diluted in filter-sterilized (0.22 μm filter) reactor effluent and 0.1 mL (of various dilutions) was spread on the medium plates. The plates were incubated inside the anaerobic (10% CO_2_, 90% N_2_) chamber, statically, at 37°C. After 6 days of incubation, 15 colonies from the plates were picked, transferred to fresh plates, and incubated for another 5 days. Afterwards, cultures were again transferred to fresh plates. The remaining biomass on the plates was used for identification using Sanger analysis (Section 2.6.2). The biomass was harvested using a loop, suspended in 0.5 mL of filter-sterilized (0.22 μm filter) PBS in DNAse free tubes (Micrewtubes^®^), centrifuged for 5 min at 20.817 g, the supernatant was removed, and the pellet was stored at −20°C until further analysis. Once the colonies on the plates of the last transfer were grown, they were inoculated in a liquid PYG modified medium (DSMZ medium 104). After fully grown, the cultures were stored anaerobically in 20% glycerol solution with Titanium (III) citrate (Ti-citrate) as a reducing agent at −80°C until their use. The identification of cultures using Sanger sequencing (Section 2.6.2) showed that all the isolates belonged to different genera of lactic acid bacteria. The isolate labelled G03 was identified as *Limosilactobacillus mucosae* and was used in further work.

As no strain of chain-elongating bacteria was obtained during the isolation campaign, *Caproicibacterium lactatifermentans* LBM19010 (Wang et al., 2022a) was purchased from the Japan Collection of Microorganisms. *Caproiciproducens lactatifermentans* (CL) and *L. mucosae* (LM) were regularly grown in the designed medium (Section 2.1.3), and the cultures were refreshed from the −80°C stock every 2 weeks for LM and monthly for CL. Furthermore, *Lacticaseibacillus rhamnosus* (LR) ATCC 7469, the type strain, was freshly grown from a −80°C stock before performing the experiments. For regular growth, 100 mL bottles with 40 mL working volume were used. The purity of strains was regularly checked by Sanger sequencing (Section 2.6.2).

#### 2.1.3 Design of Basic Glucose Medium (BGM)

To enable precise control over the dosed nutrients, an anaerobic Basic Glucose Medium (BGM) was designed. The medium was a combination of the reactor medium (Supplementary Table S1) and a widely used PYG modified medium (DSMZ medium 104). Various iterations of the medium were tested such as the addition of minerals and vitamins, the mass ratio between glucose and yeast extract, the glucose concentration, and the addition of organic acid buffer (Supplementary Table S2). The final medium was prepared as described in the Supplementary Material (Supplementary Section 1.1), and contained, per liter: 2 g K_2_HPO4, 0.001 g resazurin, 40 mL PYG salt solution (without K_2_HPO_4_), 0.5 g Cysteine-HCl.H_2_O, 0.2 mL vitamin K_1_, 1 mL of Trace element solution SL-10, 1 mL of Selenite-tungstate solution, 1 mL of 7-vitamin solution and various concentration of glucose, yeast extract, tryptone, acetate and caproic acid, depending on the experiment performed (Table 1). Additionally, 0.1 M organic acid was used as a buffer to maintain the pH at 5.5; either 11.81 g/L of succinic acid (BGM-SUC) or 8.20 g/L of sodium acetate (BGM-AC) was added (Table 1). Ti-citrate (0.05 mL/bottle) was used as a reducing agent and was added immediately prior to the inoculation.

**TABLE 1:** Overview of experiments and medium compositions.

Experiment	Organism(s)	Buffer (medium)	Glucose (g/L)	Yeast extract (g/L)	Tryptone (g/L)	Acetate (g/L)	Caproic acid (g/L)
Growth rates and biomass yields	*L. mucosae*, *C. lactatifermentans*	Succinic acid (BGM-SUC)	2	0.2	4	0.2	0
K_S_ of glucose (Low Tryptone)	*L. mucosae*	Succinic acid (BGM-SUC)	0–2	0–0.2	0–0.8	0.3	0
K_S_ of glucose (High Tryptone)	*L. mucosae*	Succinic acid (BGM-SUC)	0–6	0–0.6	0–12	0–0.6	0
K_S_ of glucose (zero-medium)	*L. mucosae*	Succinic acid (BGM-SUC)	0	0	0	0	0
Caproic acid inhibition	*L. mucosae*, *L. rhamnosus*	Acetic acid (BGM-AC)	2	0.2	4	6.2	0–12

### 2.2 Determination of specific growth rates and biomass yields

Kinetic and yield experiments were performed in triplicate in 250 mL serum bottles with 100 mL of medium and nitrogen gas in the headspace. The cultures were grown in a BGM-SUC medium (Table 1). The inoculum was grown until the early stationary phase; 10% of the medium volume was used for the inoculation (i.e. 10 mL). After the inoculation, the culture was sampled for the determination of OD_600_, pH, and initial substrate and product concentrations (Section 2.5.1). The overpressure in the bottles was corrected to around 10 kPa and the bottles were incubated on a shaker (120 rpm) at 37°C. The amount of biomass was continuously monitored using a CGQuant System (Aquila biolabs GmbH, Germany), which records the backscattered light of a laser illuminating the culture. The experiments were terminated when cultures reached the stationary phase. First, the overpressure was measured, and a gas sample was taken to determine the composition of the produced gases (Section 2.5.1). Afterwards, a subsample was filter-sterilized (0.22 µm filter) for the subsequent analysis of product concentrations. The pH, conductivity, and OD_600_ were determined in the rest of the non-filtered culture volume, which was later used for the determination of the Volatile Suspended Solids (Section 2.5.2). Furthermore, a sample of culture was taken and stored in DNAse-free tubes (Micrewtubes^®^) to check for the purity of the cultures using Sanger sequencing (Section 2.6.2). All the samples were stored at −20°C until their analysis.

### 2.3 Determination of glucose half-saturation constant (K_S_)

Experiments were performed in 96-well polystyrene plates inside an anaerobic (10% CO_2_, 90% N_2_) chamber (GP-Campus, Jacomex, TCPS NV, Rotselaar, Belgium) with a final working volume of 200 µL. BGM-SUC medium was used where the mass ratio of glucose to yeast extract was fixed to 1: 0.1, and glucose concentration was varying between 0 and 6 g/L. Two experiments were performed with different tryptone and acetate concentrations (Table 1). In the High Tryptone experiment, the mass ratio of glucose, tryptone, and acetate was fixed at 1: 2: 0.1, respectively. In the Low Tryptone experiment the acetate concentration was constant and the mass ratio of glucose: tryptone was set at 1: 0.4 (Table 1). The tubes with 2 mL of each medium were prepared, to which 10 µL of Ti-citrate was added as a reducing agent. The medium was distributed in the wells (180 µL/well). A bottle of LM culture was grown in a BGM-SUC medium until the early stationary phase and taken inside the anaerobic chamber. There, the culture was centrifuged (5,000 g, 8 min) and washed once with a medium that contained no glucose, yeast extract, tryptone, or acetate (zero-medium) (Table 1), to remove all the leftover carbon and nitrogen sources from the inoculum. The washed culture was diluted 2.5-times in the zero-medium and then used as the inoculum (20 µL/well). Each experimental condition was performed in quadruplicate. Furthermore, for each condition a negative control was prepared, where 200 µL of medium was incubated without the inoculation, to confirm the sterility of work. The growth of cultures was monitored with an automated live imaging microscope (oCelloScope, BioSense Solutions ApS, Denmark).

### 2.4 Determination of caproic acid inhibition on growth

The experiments were performed in 96-well polystyrene plates inside the anaerobic (10% CO_2_, 90% N_2_) chamber (GP-Campus, Jacomex, TCPS NV, Rotselaar, Belgium) with a final working volume of 200 µL. BGM-AC medium with varying caproic acid concentration was used. For LM growth at the following CA concentrations was tested (g/L): 0, 2, 4, 6, 7, 8, 9, 10, 11, 12. For LR growth at the following CA concentrations was tested (g/L): 0, 1, 2, 3, 4, 5, 6, 8, 10, 12. Bottles of LM and LR cultures were grown in a BGM-AC medium until the early exponential phase and taken inside the anaerobic chamber. In one of the conditions, the LM was acclimated to 4 g/L of CA (LM-4) which was added to the BGM-AC medium. When no CA was added, the labels LM-0 and LR-0 were used. The cultures were transferred at least three times in the same medium before the start of the experiment. Tubes with 2 mL of each medium were prepared, to which 10 µL of Ti-citrate was added as a reducing agent. The medium was distributed in the wells (180 µL/well), and 10% of the inoculum was added (20 µL/well). Each experimental condition was performed in quadruplicate. Furthermore, for each condition a negative control was prepared, where 200 µL of medium was incubated without the inoculation, to confirm the sterility of the work. The growth of the cultures was monitored with an automated live imaging microscope (oCelloScope, BioSense Solutions ApS, Denmark).

### 2.5 Analytical methods

#### 2.5.1 Chemical analyses

Glucose and lactate concentrations were determined by High-Performance Liquid Chromatography (HPLC). Samples are analyzed on a Shimadzu Prominence LC-2030C Plus with an RI detector (RID-20A). An isocratic method of 15 min on a Rezex ROA-Organic Acid H+ (8%) column (Phenomenex—part number 00F-0138-K0 + SecurityGuard Cartridge Kit (KJ0-4282) + SecurityGuard Cartridges Carbo-H 4 × 3.0 mm ID (AJ0-4490)) was used. The mobile phase was 5 mM sulfuric acid (Chem-lab CL00.2653.0050). The column temperature was 60°C and the temperature of samples was 4°C. The detection of glucose was done with an RI detector and the detection of lactate with a UV detector (210 nm).

Carboxylic acids (acetate, propionate, butyrate, iso-butyrate, valerate, iso-valerate, and caproate) were determined by gas chromatography (GC) with flame ionization detection (FID) as described by Candry et al. (2020) after the extraction of acids into diethyl ether as described by Andersen et al. (2014).

The gaseous head space composition was analyzed using a Compact Gas Chromatograph (Global Analyser Solutions, Breda, Netherlands), equipped with a Molsieve 5A pre-column and Porabond column (CH_4_, O_2_, H_2,_ and N_2_), and a Rt-Q-bond pre-column and column (CO_2_). Concentrations of gases were determined using a thermal conductivity detector.

#### 2.5.2 Biomass analysis

Volatile Suspended Solids (VSS) concentrations in the samples were quantified according to the Standard Methods 2540E (APHA, 1992). Briefly, glass-fiber filters were prepared by incinerating them in a muffle furnace (Naberthem GmbH, Germany) at 550°C for at least 90 min. The samples were thawed and homogenized by vortexing. A fixed volume of sample (*V*) was filtered over a glass-fiber filter under the vacuum. After the filtration, the filters were allowed to dry in the oven (Memmert, Germany) at 105°C for at least 2 h and weighed (*m*_*1*_). Afterwards, the filters were incinerated at 550°C for 90 min in a muffle furnace after which they were weighed again (*m*_*2*_). All the samples were analyzed in duplicate. The VSS concentration was calculated using the (Eq. 1).

$$VSS=\frac{\left(m_{2}-m_{1}\right)}{V}$$ (1)

### 2.6 Molecular methods

#### 2.6.1 Community structure analysis

For 16S rRNA gene amplicon sequencing, 1 mL of granular biomass was stored in a DNAse free tube (Micrewtubes^®^), centrifuged for 5 min at 20.817 g, the supernatant was removed, and the remaining pellets were stored at − 20°C until the DNA extraction. DNA was extracted according to Vilchez-Vargas et al. (2013), without the additional column purification step. 10 μL genomic DNA extract was sent out to LGC Genomics GmbH (Berlin, Germany) for library preparation and sequencing on an Illumina Miseq platform with v3 chemistry with the primers 341F (5′-CCT ACG GGN GGC WGC AG −3′) and 785Rmod (5′-GAC TAC HVG GGT ATC TAA KCC-3′) (Klindworth et al., 2013). Read assembly and cleanup was largely derived from the MiSeq SOP described by the Schloss lab (Schloss et al., 2009; Kozich et al., 2013). In brief, Mothur (v.1.44.3) was used to assemble reads into contigs, perform alignment-based quality filtering (alignment to the mothur-reconstructed SILVA SEED alignment, v. 138), remove chimeras (vsearch v2.13.3), assign taxonomy using a naïve Bayesian classifier (Wang et al., 2007) and SILVA NR v138 and cluster contigs into OTUs at 97% sequence similarity. All sequences that were classified as Eukaryota, Archaea, Chloroplasts, and Mitochondria were removed. Also, if sequences could not be classified at all (even at (super)Kingdom level) they were removed. For each OTU representative sequences were picked as the most abundant sequence within that OTU. Before subsequent analysis, absolute singletons were removed from this dataset. No rarefaction was applied before the analysis of the filtered data.

#### 2.6.2 Isolate identification

DNA was extracted according to Vilchez-Vargas et al. (2013), without the additional column purification step. The 16S rRNA gene of isolate culture was amplified by Polymerase Chain Reaction (PCR) using 27F_LGC (5′-GAGTTTGATCMTGGCTCAG341F5′-) and 1492R_LGC (5′-GGYTACCTTGTTACGACTT-3′) primer pair. Each PCR amplification mixture was prepared using a Fermentas PCR Kit, according to the manufacturers’ specifications (Thermo Fisher Scientific, Waltham, MA, United States), and PCR was carried out in a BioRad T100TM Thermal Cycler (Applied Biosystems, Foster City, CA, United States). PCR-products were purified with the QIAquick PCR Purification Kit (Qiagen, Venlo, Netherlands). The quality of the obtained purified PCR-products was checked by agarose gel electrophoresis (2% agarose gel for 30 min at 100 V). Subsequently, purified PCR-products were sent for molecular identification by bi-directional Sanger sequencing to LGC Genomics GmbH (Berlin, Germany). Forward and reverse 16S rRNA gene Sanger reads were aligned using the BioEdit package (version 7.0) to generate consensus sequences which were blasted in the National Center for Biotechnology Information (NCBI) database against the 16S rRNA gene sequence database (Madden et al., 1996; Hall, 1999).

#### 2.6.3 Phylogenetic tree construction

The phylogenetic tree was constructed using the (partial) 16S rRNA sequences obtained i) during the community analysis, ii) isolate identification, and iii) available 16S rRNA sequence references in the NCBI database. First, all the 16S rRNA sequences were aligned in the BioEdit package (version 7.0) using the function ClustalW Multiple alignment (default settings) (Hall, 1999). Afterwards, the MEGA11 software was used to construct the phylogenetic tree applying the Maximum Likelihood Tree function (default settings) (Tamura et al., 2021).

### 2.7 Data analysis and calculations

#### 2.7.1 Growth parameters

The amount of biomass was monitored with either CGQuant (Section 3.2) or oCelloScope (Section 2.3, Section 2.4) device. In the case of the CGQuant device, the raw backscatter values without any data pre-processing were directly used to construct the growth curves. In the case of the oCelloScope device, the raw images of each well were first manually checked to confirm the growth (Supplementary Video S1). Afterwards, the UniExplorer (version 11.0.1.8353) software was used to first transform the images into the Total Absorption (TA) values using the Total Absorption algorithm, which is designed as an equivalent of the OD measurements (Pontius, 2019). The TA values were used to construct the growth curves. The growth curves obtained were further processed with MATLAB (version 9.11) to determine the growth rates, the half-saturation constant, and the caproic acid growth inhibition concentrations.

To describe growth, the Gompertz model was used as in (Zwietering et al., 1990) (Eq. 2).

$$\frac{OD}{{OD}_{0}}=y=A\cdot e^{-e^{\frac{\mu_{m}\cdot e\cdot \left(\lambda -t\right)}{A}+1}}$$ (2)

The kinetic parameters describing growth in this model (i.e., A, µ, and λ) were estimated by minimizing the root squared mean deviation (RSMD) between the experimental data (i.e., y_Exp_) and the model data when mimicking the experimental growth curves (Eq. 3).

$$RMSD=\sqrt{\sum_{i=1}^{m}{\left({\hat{y}}_{i}\left(\theta \right)-y_{i}\right)}^{2}}$$ (3)

where y is the biomass concentration (OD) determined via backscatter or TA values normalized to the initial value (OD_0_), A is the maximum optical density, µ_m_ is the maximum specific growth rate of the experiment, λ is the lag time and $\hat{y}$ is the simulated normalized biomass concentration at the *ith* time point.

The determination of the half-saturation constant (K_S_) was done by fitting the µ values at different initial substrate concentrations to the Monod equation (Eq. 4).

$$µ={µ}_{\max}\;\left(\frac{S}{K_{S}+S}\right)$$ (4)

When caproic acid growth inhibition was determined, the growth rates were normalized using the average growth rate value of the four replicates determined at 0 g/L of caproate. Then, the caproic acid growth inhibition was determined by using the linear inhibition model (Eq. 5) as described in Hinshelwood (1946) and Candry et al. (2018).

$$µ={µ}_{\max}\;\left(1-K\cdot P\right)$$ (5)

In both cases (i.e., K_S_ and K), the parameter estimation was done by minimizing the RMSD between the model and experimental data as previously described. To ensure a robust estimation of the parameters and avoid the model getting stuck as local minima, the estimation of the parameters follows a bootstrap procedure to determine the value and uncertainty of the estimated parameters followed by uncertainty propagation using a Monte Carlo procedure. This procedure is described in more detail in (Regueira et al., 2021).

#### 2.7.2 Biomass yields

The biomass yields were calculated based on the biomass concentration measurements using the VSS method (Section 2.5.2) and glucose concentrations as determined with HPLC (Section 2.5.1). The amount of VSS added by inoculum was subtracted from the measured VSS in the sample (∆ Biomass concentration). The change in glucose concentration (∆ Glucose concentration) was calculated by subtracting the end glucose concentrations from the initial glucose concentrations.

The biomass yields (Y_X/S_) were calculated using Eq. 6

$$Y_{X/S}=\frac{∆Biomass\;concentration}{∆Glucose\;concentration}$$ (6)

#### 2.7.3 Statistics

To assess the significance of growth rate decrease in the presence of CA, the growth rates determined at various CA concentrations were compared to the growth rate determined at 0 g/L of caproic acid. Welch’s unequal variances *t*-test was performed in the RStudio statistical environment (version 2023.6.0.421) (R Core Team, 2023; Posit Team, 2023).

## 3 Results

### 3.1 Microbial community of granules is composed of two main functional groups—LAB and CEB

**FIGURE 1:**
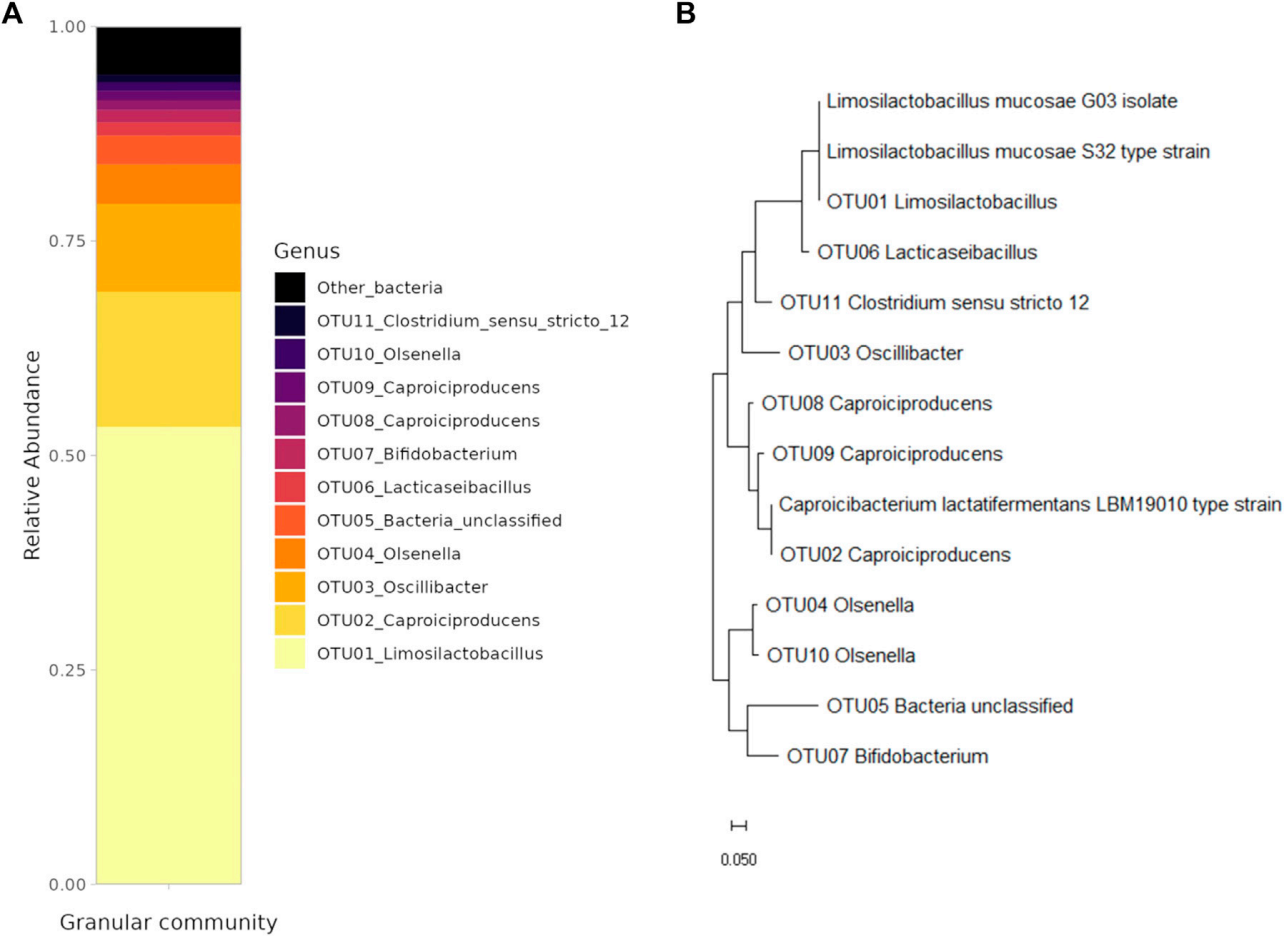
**(A)** Structure of the granular community (only the OTUs with the abundance higher than 1% are shown). **(B)** Phylogenetic tree of the granular community members, the isolate and the type strains.

An EGSB reactor was used for the production of caproic acid from a synthetic medium supplemented with glucose as a carbon source. First, the granular community was characterized using amplicon sequencing to identify present community members. Afterwards, representatives of the two most abundant OTUs were obtained for further characterization of growth in axenic cultures. The average effluent concentrations of the main organic acids produced during the 45 days of reactor operation were 2.64 ± 1.54 g/L of acetate, 1.17 ± 0.24 g/L of butyrate, 0.65 ± 0.27 g/L of iso-butyrate and 2.14 ± 0.53 g/L of caproic acid. Alongside, propionate, valerate, and iso-valerate were produced in concentrations lower than 0.3 g/L (Supplementary Figure S1). The amplicon sequence analysis of the granular community was performed on day 20 of the reactor operation (Figure 1). The community analysis showed that the community was highly enriched in two functional groups, namely, lactic acid bacteria (LAB) and chain-elongating bacteria (CEB). The LAB from the Lactobacillaceae family (e.g., OTU01, OTU06) composed 56.1% of the community, whereas the OTU01_*Limosilactobacillus* on its own represented 53.3% of the community. Another LAB present were the members of the *Olsenella* genus (e.g., OTU04, OTU10) composing 6.3% of the community. The CEB belonging to the *Caporiciproducens* genus (e.g., OTU02, OTU08, OTU09) formed 20.6% of the community with the most abundant OTU02_*Caproiciproducens* representing 15.8% of the community. The third most abundant OTU03 belonged to the *Oscillibacter* sp. (10.2%).

The representatives of LAB and CEB were obtained either by isolation or from the culture collection. A strain of *Limosilatobacillus mucosae* labelled as G03 was isolated using the isolation by plating technique as described in Section 2.1.2. The strain has a 100% match in the v3–v4 region (445 bp) of the 16S ribosomal sequence to the most abundant *Limosilactobacillus* (OUT01) in the system, as can be seen from the constructed phylogenetic tree (Figure 1). As only LAB were isolated with the applied approach, a representative of CEB bacteria, the strain of *Caproicibacterim lactatifermentans* (Wang et al., 2022a) was ordered from the Japan Collection of Microorganisms. This strain has an identical v3–v4 region (445 bp) of 16S ribosomal sequence as the most abundant representative from the *Caproiciproducens* genus (OTU02) in the system (Figure 1). Despite the difference in the genus name, 100% similarity in the sequence indicates that the two bacteria could belong to the same genus.

### 3.2 Growth of LAB and CEB in the newly designed medium

**FIGURE 2:**
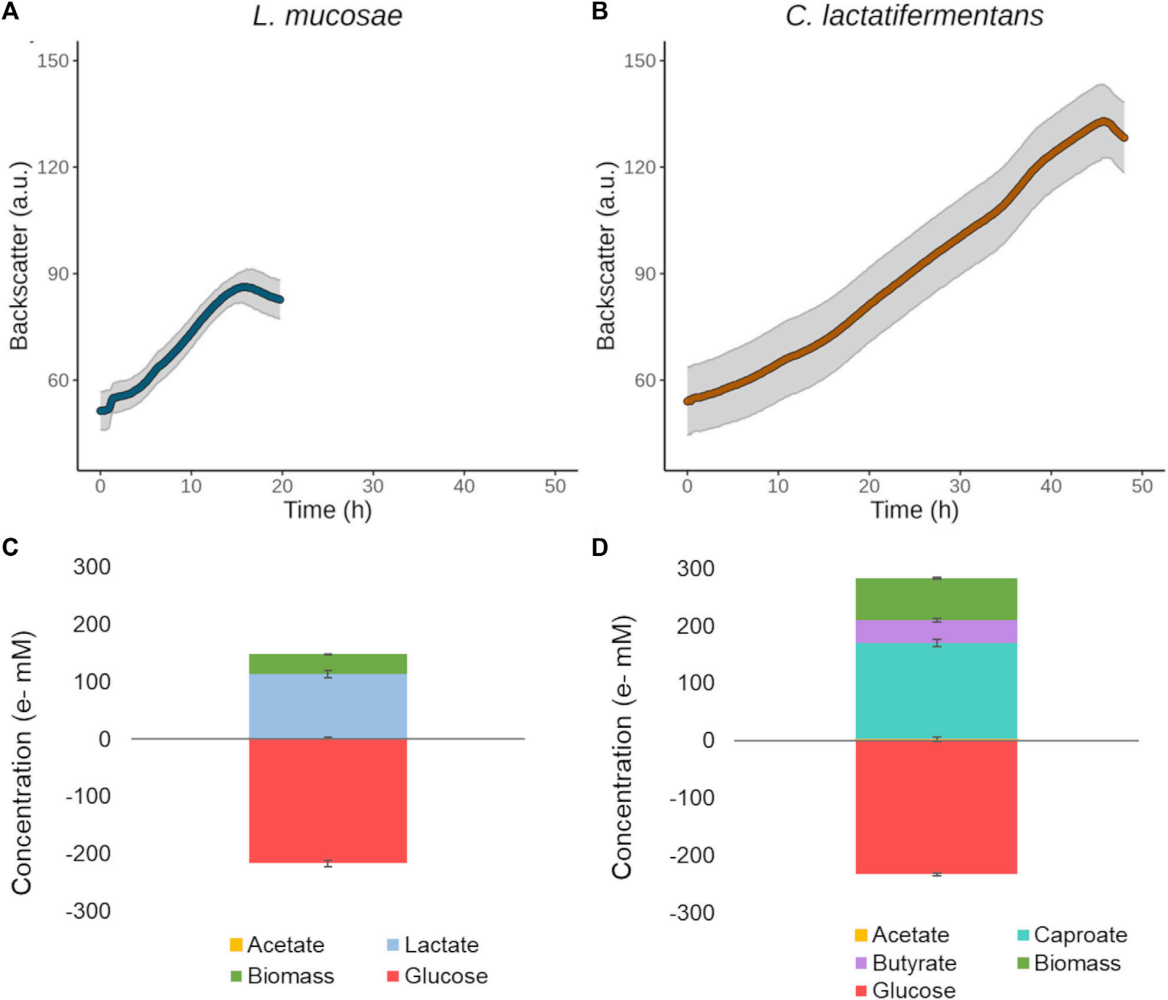
Growth in the constructed BGM-SUC medium with 2 g/L of glucose. **(A)** *L. mucosae* G03 growth curve. **(B)** *C. lactatifermentans* growth curve. **(C)** Electron balance for *L. mucosae* G03. **(D)** Electron balance for *C. lactatifermentans*.

To be able to characterize LAB and CEB for their growth in pure cultures, a Basic Glucose Medium (BGM) was designed that enabled precise control over the provided nutrients. Different iterations of the medium were tested, such as the addition of minerals and vitamins, the mass ratio between glucose and yeast extract, the glucose concentration, and the organic acid buffer used (Supplementary Table S2). Based on the growth of cultures and change in pH (Supplementary Table S2) the final medium was decided. The medium contained minerals, vitamins, 0.1 M organic acid buffer (acetate or succinate); and glucose, yeast extract, tryptone, and acetate in the mass ratio of 1: 0.1: 2: 0.1, respectively. The strains were unable to grow in the medium without added glucose, showing that the provided yeast extract, tryptone, acetate, and organic acid buffer alone could not serve as a carbon source (Supplementary Figure S2). Both bacteria grew well in the BGM medium with succinate as a buffer (BGM-SUC), which can be seen from the short lag phase present during the growth (Figure 2). Furthermore, the isolated strain *L. mucosae* G03 produced 0.78 g/L of lactate from glucose (2 g/L), and the concentration of acetic acid remained stable throughout the growth (0.22 ± 0.01 g/L at the start and 0.24 ± 0.01 g/L at the end of growth), but the electron balance showed that not all the fermentation products were measured (Figure 2). The main product of *C. lactatifermentans* was caproic acid (0.61 ± 0.02 g/L), which was accompanied by some butyric acid production (0.17 ± 0.01 g/L). The concentration of acetic acid also remained stable (0.22 ± 0.01 g/L at the start and 0.25 ± 0.02 g/L at the end of experiments), and no odd-chain carboxylic acid products were detected. The closed electron balance indicates that all the products were determined (Figure 2).

### 3.3 LAB have higher growth rates but lower biomass yields than CEB

The main growth parameters [i.e., growth rate (µ), biomass yields (Y_X/S_), and glucose half-saturation constant (K_S_)] were determined for the axenic cultures of *L. mucosae* and *C. lactatifermentans* grown in BGM-SUC medium. The growth rates were estimated with the Gompertz model, where a good fit between the data and equation was observed (Supplementary Figure S3). The growth rate of *L. mucosae* culture in the serum bottles was 0.051 ± 0.005 h^−1^, which is twice as much as that of *C. lactatifermentans* (0.026 ± 0.004 h^−1^). On the contrary, the biomass yields determined through VSS were double for the culture of *C. lactatifermentans* (0.239 ± 0.007 g biomass/g glucose) compared to the biomass yields of *L. mucosae* (0.120 ± 0.005 g biomass/g glucose) (Table 2).

**TABLE 2:** Growth parameters for *L. mucosae* G03 and *C. lactatifermentans.*

	µ (h^−1^)	Y_X/S_ (g biomass/g glucose)	K_S_ for glucose (g/L)
*L. mucosae* G03	0.051 ± 0.005	0.120 ± 0.005	0.35 ± 0.05
*C. lactatifermentans*	0.026 ± 0.004	0.239 ± 0.007	NA

**FIGURE 3:**
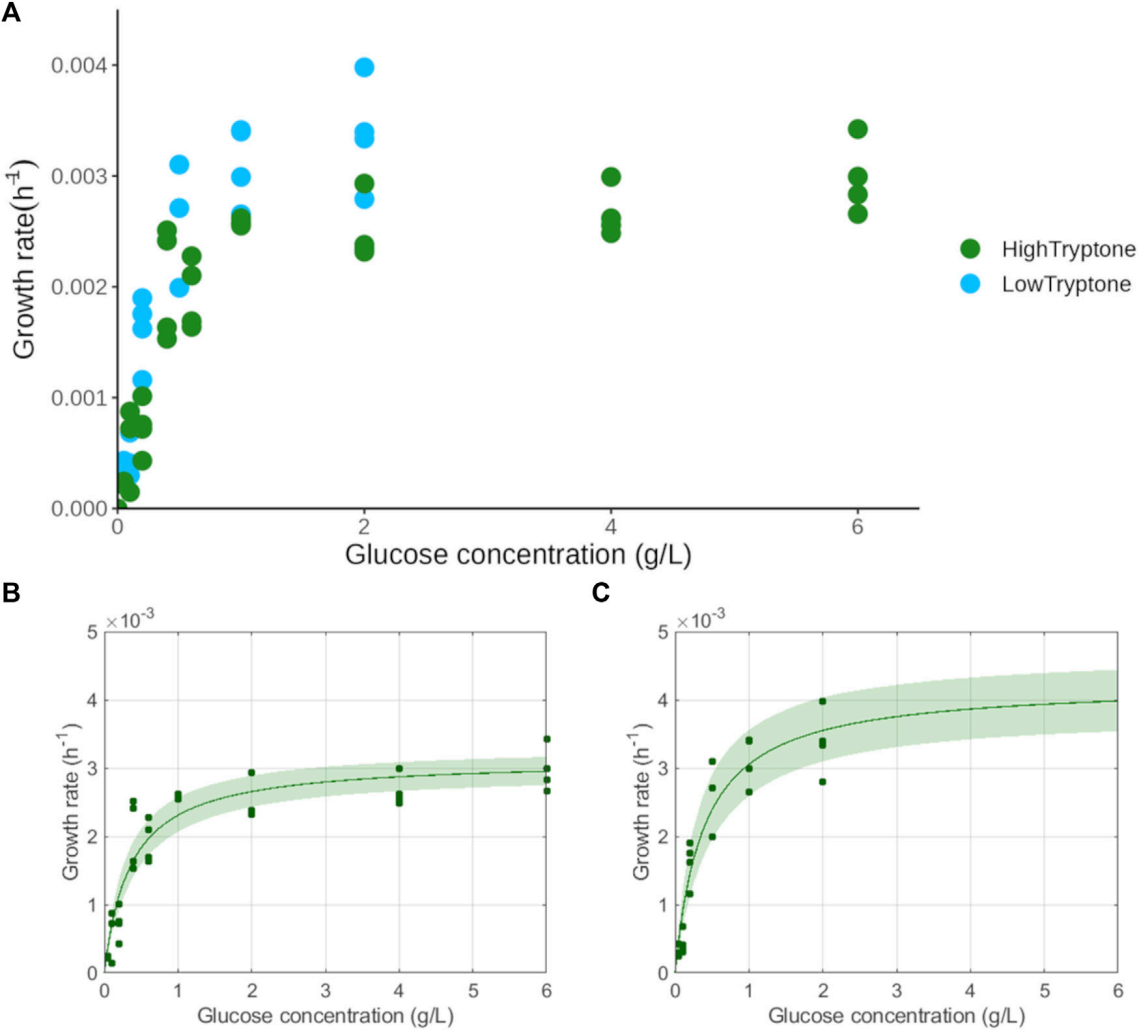
Determination of half-saturation constant of glucose for *L. mucosae* G03. **(A)** Growth rates in the function of initial glucose concentration for two experiments with different concentrations of tryptone. **(B)** Fit of the Monod equation in the High Tryptone experiment. **(C)** Fit of the Monod equation in the Low Tryptone experiment.

To determine the K_S_ of glucose for *L. mucosae*, the culture was grown in 96-well at varying initial glucose concentrations and at two different tryptone concentrations (Section 2.3). The growth rates were estimated with the Gompertz model (Supplementary Figure S3) and the K_S_ values were further obtained by fitting the Monod equation to the data (Figure 3). The estimated Ks of glucose was similar in both media where tryptone was added in different concentrations. The K_S_ determined in the Low Tryptone experiment was 0.38 ± 0.07 g/L glucose and that determined in the High Tryptone experiment was 0.35 ± 0.05 g/L glucose. Here, the two datasets are provided to show the robustness of the method. The growth was monitored at very low glucose concentrations (e.g., 0.05 g/L) which resulted in a low increase of biomass, difficult to detect with the traditional biomass monitoring methods (e.g., OD_600_). The approach applied in this work has not been used in the literature so far. Repeating the experiment in two similar, and at the same time different conditions, ensures the robustness and reliability of the results obtained. The K_S_ value obtained in the High Tryptone experiment was selected for further discussion (Table 2), as the same growth medium was also used in the experiments for the determination of growth rates and biomass yields (Table 1). The culture of *C. lactatifermentans* was unable to grow in a 96-well plate so the determination of K_S_ was not possible.

### 3.4 Caproic acid inhibits LAB growth, but acclimation seems to enhance their resilience

The main product of interest in the reported bioprocess is caproic acid (CA), which is known to inhibit microbial growth. To understand the impact of CA on the growth of *L. mucosae* isolate, the response of non-acclimated (LM-0) and acclimated *L. mucosae* to 4 g/L of CA (LM-4) to the media containing increasing concentration of CA was tested. Besides, the inhibitory effect of CA was tested on the culture of *L. rhamnosus* type strain (LR-0), which was not acclimated to CA, to evaluate if the native environment plays a role in the tolerance to CA.

**FIGURE 4:**
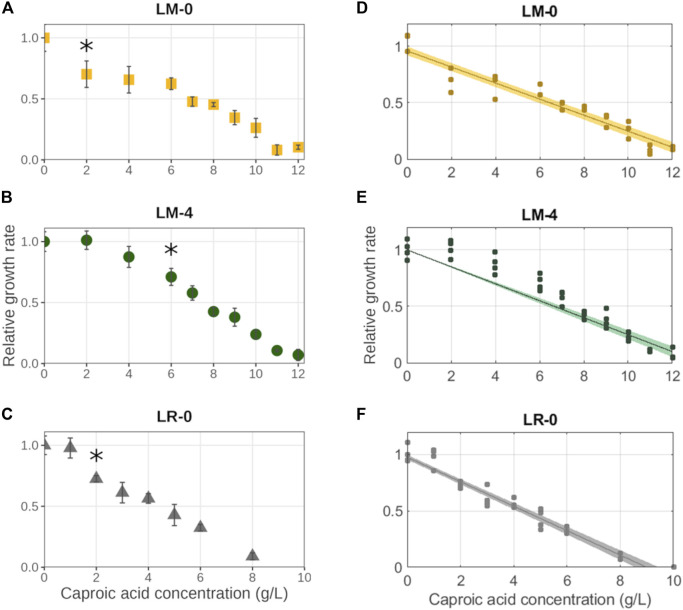
Impact of caproic acid on the growth rates of three different cultures. **(A–C)** Average values are shown with error bars representing standard deviations. The asterisks (*) indicate the caproic acid concentration at which growth rate was significantly decreased compared to the growth rate at 0 g/L of caproic acid. **(D–F)** The fit of linear model with confidential interval is shown. **(A–D)** isolate *L. mucosae* G03 (LM-0) (*n* = 3). **(B, E)** isolate *L. mucosae* G03 adjusted to 4 g/L of CA (LM-4) (*n* = 4). **(C, F)** type strain of *L. rhamnosus* (LR-0) (*n* = 4).

In all the experiments, the growth inhibitory effect of CA showed a linear trend where the growth rates were decreasing with the increasing CA concentration (Figure 4). On the contrary, the duration of the lag phase increased with increasing caproic acid concentration in both experiments with *L. mucosae*, but not in the experiment with *L. rhamnosus* (Supplementary Figure S4). In both experiments with *L. mucosae*, the cultures were able to grow at the highest CA concentrations tested (12 g/L). The linear model predicted that the growth would no longer occur at 13.6 ± 0.5 g/L and 13.1 ± 0.3 g/L of CA for LM-0 and LM-4, respectively (Table 3). The acclimation of inoculum enabled higher growth rates at low CA concentration but did not significantly improve the tolerance of this strain to high CA concentrations. A significant decrease in growth rate (compared to the growth at 0 g/L of CA) only occurred at 6 g/L of CA in the LM-4 experiment, while the growth rates already significantly decreased at 2 g/L in the LM-0 experiment (Figure 4). Similarly, the growth rates were significantly lower at 2 g/L in the LR-0 experiment. Overall, the *L. rhamnosus* is more suspectable to the inhibitory effects of the CA as no growth was present at 10 g/L of CA and the model predicted the growth inhibition at 8.9 ± 0.3 g/L (Table 3), which is lower than the values in the *L. mucosae* experiments.

**TABLE 3:** Caproic acid growth inhibition concentration as determined by the model for three different cultures. The average values and standard deviations are provided. (LM-0: isolate *L. mucosae* G03 (*n* = 3); LM-4: isolate *L. mucosae* G03 adjusted to 4 g/L of CA (*n* = 4); LR-0: type strain of *L. rhamnosus* (*n* = 4)).

Culture	Inhibitory total CA concentration (g/L) [concentration in mM]	Inhibitory protonated CA concentration (g/L) [concentration in mM]
LM-0	13.6 ± 0.5 [117 ± 4]	2.6 ± 0.1 [22.7 ± 0.8]
LM-4	13.1 ± 0.3 [113 ± 3]	2.5 ± 0.1 [21.8 ± 0.5]
LR-0	8.9 ± 0.3 [76.6 ± 2.6]	1.7 ± 0.1 [14.8 ± 0.5]

## 4 Discussion

### 4.1 LAB and CEB have different growth strategies

A continuous bioprocess for high-rate production of caproic acid from sugars is an engineered environment where the substrate turnover is high and a mildly-acidic pH is maintained stable. This creates a unique environment for microorganisms to thrive and interact. The community of our system was mainly composed of LAB belonging to the Lactobacillaceae family or *Olsenella* genus, and CEB from the *Caproiciproducens* genus, altogether representing 83.0% of the community. Similarly, other granular sludge systems for the production of CA reported the co-existence of these two functional guilds (Table 4). Furthermore, LAB are commonly found in the systems for continuous production of CA (Candry and Ganigué, 2021). Both LAB and CEB can use glucose as a substrate, generating various end products and conserving different levels of energy (i.e., via ATP) from it (Table 5). The members of the *Limosilactobacillus* genus are known for their heterofermentative metabolism, although there is little information available on the product profile of the different species within the genus. The isolate G03 was identified as a strain of *L. mucosae* based on the 16S ribosomal gene sequence (Figure 1). In particular, the species *L. mucosae* is reported to be obligately heterofermentative (Roos et al., 2000) and the annotated genome of *L. mucosae* LM1 (Lee et al., 2012) available in the Kyoto Encyclopedia of Genes and Genomes (KEGG) (Kanehisa et al., 2023) shows that the strain can produce lactate, ethanol, and acetate from sugars. In this study, the main product of glucose fermentation by *L. mucosae* G03 was lactate, although the disability of closing the electron balance (Figure 2) indicates that other compounds are produced. Furthermore, the strain produced CO_2_ (Supplementary Table S3), which can be released in the phosphoketolase pathway, indicating that ethanol could be produced besides lactate (Spector, 2009). The culture collection strain of *C. lactatifermentans* (Wang et al., 2022a) was considered a good model organism for the CEB in our system due to its high similarity of the 16S ribosomal gene to the most abundant *Caproiciproducens* sp. (Figure 1). The strain is known to produce acetate, butyrate, and caproate from glucose and lactate using the reverse β-oxidation pathway. The ratio between products depends on the provided ratio between the electron donor (e.g., glucose, lactate) and the electron acceptor (e.g., acetate) (Wang et al., 2022b). In this work, the mass ratio between glucose and acetate was high (i.e. 10), and the strain mainly produced caproate (Figure 2).

**TABLE 4:** Overview of granular systems operated with glucose or complex substrate. When available, the exact concentrations and community data are provided from the indicated reference period of reactor operation.

Substrate	Produced organic acids (concentration in g/L)	Primary fermentation	Secondary fermentation	References
Synthetic medium with glucose	Lac (≤24), Ac	*Bacillus laevolacticus* (axenic culture)	Not present	De Boer et al. (1990)
Synthetic medium with glucose	Ac, Prop, But, Val	*Olsenella* sp., *Lactobacillus* sp., *Clostridium pasterianum*	Not reported	Tamis et al. (2015)
Synthetic medium with glucose	Lac (19–20)	*Lactobacullus* sp., *Leuconostoc* sp.	Not reported	Kim et al. (2016)
Solids-free thin stillage	Ac, Prop, But, Val, Cap (4.9), Hep, Oct	*Olsenella* sp. (Coriobacteriaceae—24%)	*Caproiciproducens* sp. and Ruminococcaceae bacterium CPB6, *Oscillibacter* sp. (Ruminococcaceae family—69%)	Carvajal-Arroyo et al. (2019)
Solids-free thin stillage	Ac (3.1), But (4.3), Cap (4.3); Prop, iso-But, Val in small amounts	*Olsenella* sp. (7%)	*Caproiciproducens* sp. (86%)	Mariën et al. (2022a)
synthetic medium with glucose	Ac (2.3), Prop (1.6), But (2.5), Cap (4.8); Val in small amounts	*Olsenella* sp. (86%)	*Caproiciproducens* sp. (9.5%)	Mariën et al. (2022b)
synthetic medium with glucose	Ac (2.6), But (1.2), iso-But (0.7), Cap (2.1); Prop, Val, iso-Val in small amounts	*Limosilactobacillus* sp. (53%), *Olsenella* sp., (5%)	*Caproiciproducens* sp. (16%)	This study

Ac, acetate; Prop, propionate; But, butyrate; iso-But, iso-butyrate; Val, valerate; iso-Val, iso-valerate; Cap, caproate; Hep, heptanoate; Oct, octanoate; Lac, lactate.

**TABLE 5:** Overview of possible pathways and energy production in chain-elongating bacteria and lactic acid bacteria.

Pathway	Stoichiometry	Number of reaction steps	Mole ATP per mole glucose	References
Chain elongation (Reverse β-oxidation)	1.5 Glucose → 1 Caproic acid + 3 CO_2_ + 2 H_2_ + 1 H_2_O	19	3.67	Mariën et al. (2022b); Wang et al. (2022b)
Homofermentative (Embden–Meyerhof–Parnas glycolysis)	1 Glucose → 2 Lactic acid	10	2	Spector, (2009)
Heterofermentative (Phosphoketolase pathway)	1 Glucose → 1 Lactic acid + 1 ethanol + 1 CO_2_	8	1	Spector, (2009)

Fermenting glucose to lactate and ethanol can yield between 1 and 2 mol of ATP per mole of glucose, depending on the pathway used (Spector, 2009). This is almost two to four times less than the generated energy in the chain-elongating metabolism where 3.67 mol of ATP are typically produced from 1 mol of glucose (Mariën et al., 2022b). Assuming a similar biomass yield on ATP, the CEB are expected to be more efficient in substrate usage and can achieve higher biomass yields compared to the LAB. Here, we have confirmed this hypothesis, as the biomass yields for *C. lactatifermentans* were approximately two times higher than those of *L. mucosae* (Table 2). On the other hand, shorter metabolic pathways run faster compared to the longer ones, when the substrate supply allows it. This enables a higher rate of energy production, which further allows for a higher growth rate of the organism using shorter metabolic pathways (Kreft et al., 2020). Thus, the pathway length is an important parameter in the growth of microorganisms. Fermentation of glucose to lactate and/or ethanol includes 8 or 10 reaction steps, while fermentation of glucose to caproate requires 19 reaction steps (Table 5). Based on the pathways length it is expected that the LAB will reach higher growth rates compared to the CEB. Here, batch cultivation was used to experimentally confirm this hypothesis, as the growth rates of *L. mucosae* were approximately two times higher than those of *C. lactatifermentans* (Table 2). Overall, the growth rates determined here are lower compared to those available in the literature. For instance, the growth rate of LAB *Lactobacillus pentosus* on glucose at pH 6.2 was determined to be 0.62 h^−1^ (Wang J. et al., 2021), and the growth rate of *Bifidobacterium adolescentis* at pH 5.5 was 0.21 h^−1^ (Amaretti et al., 2007), which are 4–12-times higher compared to the determined growth rate of isolated *L. mucosae* (0.051 h^−1^). Furthermore, the growth rate of *C. lactatifermentans* (0.026 h^−1^) observed in this work was 4-times lower than that of *Caproicibacter fermentans,* where the measured growth rates with fructose as a substrate were 0.1 and 0.11 h^−1^ at pH 5 and pH 6, respectively (Flaiz et al., 2020). However, the growth rate of *C. lactatifermentans* was similar to that determined for *Caproiciproducens* sp. 7D4C2, where the growth rate of 0.021 h^−1^ was observed for cultures grown with fructose at a pH of 5.5 (Esquivel-Elizondo et al., 2021). Lower growth rates could be explained by the choice of medium, which was designed in a way that it represents the conditions in a continuous bioprocess for the production of MCCA (Section 2.1.3), and not in the way that it optimally supports the growth of studied organisms.

Altogether, the two functional guilds studied here (LAB and CEB) seem to follow distinct ecological strategies in their growth. LAB with their high growth rates and low biomass yields resemble the behavior of copiotrophs (r-strategists) which are known to thrive in substrate-rich environments. They are characterized by fast growth, inefficient substrate usage, and low affinity for the substrate. On the contrary, the growth of CEB resembles that of an oligotroph (K-strategist) which thrives in substrate-limiting environments. K-strategists are known for their slow growth, but efficient substrate use and high affinities for the substrate (Andrews and Harris, 1986; Klappenbach et al., 2000; Yin et al., 2022). Comparing the growth rates and biomass yields of the two organisms confirms the assumption of r- and K-strategists. Furthermore, the available genetic information is in agreement with this theory, as the r-strategists are expected to encode a large number of *rrn* operons, which enables them to produce a large number of ribosomes and consequently grow faster. On the contrary, K-strategists usually encode a low number of *rrn* operons, as fast production of ribosomes is not important for their slow growth (Klappenbach et al., 2000). The annotated genome of *L. mucosae* LM1 encodes 8 *rrn* operons (Lee et al., 2012), compared to the 3 *rrn* operons encoded in the genome of *C. lactatifermentans* (Wang H. et al., 2021). Alongside, the determined K_S_ value of glucose for *L. mucosae* was 0.35 g/L which is in the range of what is available in the literature, although the reported values vary greatly. For instance, the K_S_ of glucose for *L. pentosus* was determined to be 0.041 g/L (Wang J. et al., 2021), for the *Lactobacillus case* the reported value was 0.35 g/L (Park et al., 2018), for *Lactobacillus casei* ssp. *rhamnosus* 0.5 g/L (Youssef et al., 2005) and for *B. adolescentis* the determined Ks of glucose was 1.57 g/L (Amaretti et al., 2007). Considering the theory, the expected value for *C. lactatifermentans* would be less than that of *L. mucosae*, but this should be experimentally confirmed.

### 4.2 LAB are tolerant to high concentrations of caproic acid

In the bioprocess where the production of MCCA takes place, CA is the most common product of interest. The MCCA are known to inhibit bacterial growth by impacting the integrity and fluidity of microbial membranes (Royce et al., 2013). Furthermore, due to their hydrophobic nature, the undissociated forms of MCCA can migrate directly into the cell where they dissociate. This causes the decrease of intracellular pH and alters the behavior of the cell by activating the plasma membrane ATPase and increasing the proton pumping capacity of the cell (Palmqvist and Agerdal, 2000). The level of these responses correlates to the concentration of undissociated MCCA, which is directly correlated with the environmental pH (Palmqvist and Agerdal, 2000). However, in the work of Candry et al. (2018), the growth inhibition of ethanol chain-elongator *Clostridium kluyveri* was observed at 91.3 ± 10.8 mM of total CA which at an experimental pH of 7.4 resulted in only 0.18 ± 0.01 mM of undissociated CA. This led to the suggestion that also the dissociated forms of CA impose inhibition on the growth of bacteria, although the mechanisms were not proposed.

The impact of end products on the growth of microorganisms is an important factor when designing a bioprocess, where parameters such as product titer and pH must be considered. The first indication of the CA toxic limit in a mixed culture MCCA-production bioprocess was reported to be 38.6 mM of total CA which at pH 5.5 resulted in 7.5 mM of undissociated CA (Ge et al., 2015). Until this work, none of the pure culture studies reached the proposed value of 7.5 mM of undissociated CA (Table 6). The highest tolerance to undissociated CA was reported for the bacterium *L. rhamnosus*, where cultures were able to grow at the highest tested concentrations with 2.9 mM of undissociated CA (Yáñez et al., 2008). In our work, the prediction of growth inhibition for *L. rhamnosus* was at 76.6 mM of total CA, which equals to 14.8 mM of undissociated CA. Furthermore, the inhibition value of isolate *L. mucosae* G03 was even higher, as the growth still occurred at the highest tested concentration of CA (103 mM). The growth inhibition was projected at the concentration of 117 mM of total CA and 22.7 mM of undissociated CA, which is the highest concentration reported so far. One explanation for the observed growth at high CA concentrations could be the choice of a highly sensitive method for biomass monitoring. Live-imaging microscopy was used (Section 2.4) which is more sensitive compared to the optical density measurements (Fredborg et al., 2013), meaning that a small increase in biomass at high CA concentrations might have been detected which was not the case in other studies.

**TABLE 6:** Overview of caproic acid concentrations at which microbial growth was inhibited.

Organism	Medium pH	Total CA concentration inhibition (mM)	Protonated CA concentration inhibition (mM)	References
Mixed community	5.5	38.6	7.5	Ge et al. (2015)
*C. kluyveri*	7.4-8.2	91.3 ± 10.8	0.18 ± 0.01	Candry et al. (2018)
*E. coli*	7	40	0.3	Royce et al. (2013)
co-culture of *C. kluyver + C. autoethanogenum*	6	12	0.88	Diender et al. (2016)
*L. rhamnosus*	Drops from 5.7 to 4.1	>20.6	>2.9	Yáñez et al. (2008)
*L. mucosae*	5.5	117	22.7	This study
*L. rhamnosus*	5.5	76.6	14.8	This study

Little is known about the role of CE and CEB in nature, and no natural environment likely reaches the CA concentration comparable to that in a bioprocess intended for its production. In this study, we were interested in investigating whether a non-native LAB closely related to our isolate but from another environment can tolerate similar levels of CA as the isolated LAB. The comparison of CA inhibition on the growth of the isolated *L. mucosae* to the growth of a commonly known type strain of the species *L. rhamnosus* isolated from the human gastrointestinal tract (Goldin et al., 1992) showed that our isolate can tolerate higher CA concentrations (13.6 g/L mM for *L. mucosae* compared to 8.9 for *L. rhamnosus*). This indicates that the microbes from an environment containing a given toxic product may have more efficient mechanisms to fight the product toxicity compared to the microorganisms from other environments. Another interesting observation is that the acclimation of culture to the bioprocess-relevant concentrations of CA (i.e., 4 g/L) did not improve the overall tolerance of the culture to CA. In both experiments (LM-0 and LM-4), the cultures were still able to grow at 12 g/L of CA, and the complete growth inhibition was projected at similar concentrations of CA, 13.0 and 13.6 g/L for LM-4 and LM-0, respectively. However, the acclimation of the culture did enable higher growth rates at low CA concentrations, indicating that once the culture is adjusted it can grow at growth rates similar to those where no toxic product is present. A short-term (3 h) pre-adaptation of *Escherichia coli* to octanoic acid showed that pre-adapted cells were significantly more resistant to the fluidizing effects of octanoic acid on the cell membrane compared to unadapted cells. On the other hand, the leakage of the membrane caused by octanoic acid was similar in both un-adapted and pre-adapted cultures (Royce et al., 2013). The pre-exposure of culture to an inhibitory product seems to enable it to better mitigate some effects, although there is limited knowledge on the mechanisms of bacterial defense towards the MCCA.

### 4.3 Implications for the bioprocess design and operation

Production of CA through CE metabolism in sugar-based or complex media is hypothesized by many authors to be a two-step process (Scarborough et al., 2018b; Nzeteu et al., 2018; Carvajal-Arroyo et al., 2019; Lambrecht et al., 2019; De Groof et al., 2020; Mariën et al., 2022a). The carbohydrates are first converted to lactate by LAB, which then serves as an electron donor for the production of CA by CEB. This conclusion is mainly made based on the fact that the LAB are present in the system, but no study directly evaluated the carbon flow in such systems. One of the most direct proofs of lactate-mediated CE was provided in the work of Carvajal-Arroyo et al. (2019), where electron donors (i.e., lactate or ethanol) were spiked directly to a chain elongation community that produced CA from a carbohydrate-rich stream (i.e., thin stillage). The production of CA only occurred in the case when lactate was provided, indicating that ethanol could not serve as the electron donor. However, this does not exclude the possibility that the carbohydrates directly served as the electron donors for the production of CA. In fact, a study by Scarborough, Scarborough et al., 2018a, that combined metatranscriptomic and thermodynamic approaches to study the community producing MCCA from the residues of lignocellulosic ethanol production, identified an OTU that likely produced CA directly from xylose, indicating that the CEB could compete for the available sugar molecules. Furthermore, the recent work by Candry et al. (2023), where labelled nitrogen was used to follow the growth of individual cells in a sugar-based granular CE system showed, that members of Oscillospiraceae may be competing for sugars with the LAB from the *Olsenella* genus under the conditions of normal bioprocess operation. Considering the high growth rates of LAB, it seems intuitive that they are more successful in consuming sugars than most other microorganisms. However, this is only true when the substrate concentrations are sufficiently high, such as in batch or fed-batch reactor systems. In a continuous system that is not overloaded, substrate concentrations in the reactor at steady state are constant and low (Liu, 2017). In a situation like this, K-strategists (i.e., CEB) tend to outcompete r-strategists (i.e., LAB) because of their higher affinity for the substrate which enables them to thrive in substrate-limited environments. This theory has been successfully confirmed in some biological wastewater treatment plants (Yin et al., 2022). Obtaining the K_S_ values of *C. lactatifermentans*, or a similar CEB, would enable to more precisely predict under which operational conditions would LAB outcompete CEB for sugars, and *vice versa*.

A granular sludge bioprocess is a unique example of a continuous production system from the kinetics and ecological point of view, as the biomass is sustained inside the bioreactor in the form of biofilm. Bacteria with growth rates below the critical retention time (e.g., CEB) or too low affinity for the substrate (e.g., LAB) are not under the selection pressure of being washed out, as is normally the case in the continuously operated systems with suspended biomass. Furthermore, the kinetics of substrate and product diffusion in a biofilm must be considered, which are different from those in suspended systems. Besides, product toxicity is an important factor for the growth of organisms, although it has been shown here that the pre-acclimation of a culture restores the growth rates to normal values. Altogether, these factors make the modeling of such systems complicated. Regardless, the microbes inside the granules are in close proximity having the opportunity to interact. Alongside the probable cross-feeding of the CEB on the lactate produced by the LAB, the two functional guilds could be exchanging other nutrients. The LAB are known for their auxotrophies for amino acids and vitamins (Teusink and Molenaar, 2017), although there is no available information on their synthesis by *L. mucosae* G03. On the other hand, based on its genome, the strain of *C. lactatifermentans* used in this study lacks the ability to produce 12 out of 19 amino acids (Wang H. et al., 2021). However, this information should be confirmed experimentally, as the same study predicted that the ethanol chain-elongator *C. kluyveri* lacks the ability to synthesize three amino acids, but this microorganism is able to grow in a chemically defined medium without added amino acids (Stadtman and Barker, 1949; Thauer et al., 1968). Nevertheless, since the LAB and the CEB are always present together, the two might need each other’s proximity. The ecological interactions between them should be further investigated.

In a granular sludge bioprocesses the biomass self-arranges to form biofilm in the shape of granules. In general, the process of any biofilm formation can be divided into three main phases, namely, biofilm initiation, maturation, and dispersion (Trunk et al., 2018). Besides bacterial cells, the main biofilm components are extracellular polymeric substances (EPS). The EPS mainly consists of polysaccharides and proteins, which both have an important role in the formation of biofilm and in providing its structural stability (Nwodo et al., 2012; Fong and Yildiz, 2015). Numerous theories have been established to explain the formation of methanogenic granules, the role of EPS, and the involvement of individual species (Hulshoff Pol et al., 2004), but little is known about the role of individual species in the formation of fermentative granules. The available studies on the fermentative granular sludge processes, where sugar or complex medium was provided, report the presence of LAB which either belong to the Lactobacillaceae family or *Olsenella* genus (Table 4). The LAB, especially the members of the Lactobacillacae family, are well-known biofilm formers, and producers of various EPS (Kubota et al., 2008; Wallis et al., 2018). For instance, the *Limosilactobacillus* (“*limosus*” means slimy) genus got its name from the property of most strains to produce exopolysaccharides from sucrose (Zheng et al., 2020). A metagenomic study showed that 16 out of 93 *L. mucosae* strains encode EPS-producing operons of the same type (Jia et al., 2020). Furthermore, at high glucose concentrations (BGM-AC medium, 5 g/L glucose, shaking) the isolate *L. mucosae* G03 self-arranged in granules of different shapes and sizes (Supplementary Figure S5), confirming that this strain is capable of EPS production. This behavior was not observed at the standard experimental conditions with lower glucose concentration (BGM-SUC medium, 2 g/L), which could be due to the lower biomass concentrations present in the system. Considering *Olsenella* sp., it has been reported that *Olsenella uli* can grow on a pure culture biofilm (De Paz et al., 2007), indicating that also members of this genus can have the ability to form biofilms. When targeting the production of longer-chain carboxylic acids, the members of the *Clostridium* or *Caproiciproducens* genus are present in the community (Table 4), where the *Clostridium* sp. are known biofilm producers (Pantaléon et al., 2014). To date, there are no reports of pure culture biofilm growth for *Caproiciproducens* sp, although this does not necessarily mean that its members are not capable of EPS synthesis and biofilm formation. In fact, filamentous, floc-like structures were observed at the end of the growth of *C. lactatifermentans* in BGM-AC medium (2 g/L of glucose, initial pH 6.3, shaking) (Supplementary Figure S5); these were not observed in the standard experimental conditions (BGM-SUC medium, 2 g/L glucose, pH 5.5, shaking). One of the theories in the formation of methanogenic granules called the “Spaghetti theory” recognizes the filamentous growth of *Methanothrix* sp. as one of the key factors of granular biofilm initiation and maturation (Hulshoff Pol et al., 2004). Further characterization of community members for their potential for biofilm formation and EPS production is required to better understand the formation of fermentative granules.

## 5 Conclusion

Mixed-culture bioprocesses are challenging to understand and model due to their complex microbiome structure. Characterization of individual microorganisms, understanding of their interactions, and understanding of the impact of environmental parameters on the bioprocess can aid in gaining control over the bioprocess. In this work, two main functional guilds in the production of caproic acid were investigated and insights into their growth were provided. The lactic acid bacteria grow faster in the substrate-rich environment compared to the chain-elongating bacteria and are tolerant to high caproic acid concentrations (e.g., 12 g/L), which enables them to persist and thrive in the bioprocess intended for caproate production. Furthermore, lactic acid bacteria might have an important role in the granular sludge systems due to their ability of biofilm formation. Nevertheless, further characterization of community members is needed before knowledge-driven steering of the microbiomes and their functionality will be possible.

## Data Availability

The datasets presented in this study can be found in online repositories. The names of the repository/repositories and accession number(s) can be found below: https://www.ncbi.nlm.nih.gov/nuccore/OR497829 and https://www.ncbi.nlm.nih.gov/bioproject/PRJNA1012877/.

## References

[B1] Agler M. T. Wrenn B. A. Zinder S. H. Angenent L. T. (2011). Waste to bioproduct conversion with undefined mixed cultures: the carboxylate platform. Trends Biotechnol. 29, 70–78. 10.1016/j.tibtech.2010.11.006 21190748

[B2] Amaretti A. Bernardi T. Tamburini E. Zanoni S. Lomma M. Matteuzzi D. (2007). Kinetics and metabolism of Bifidobacterium adolescentis MB 239 growing on glucose, galactose, lactose, and galactooligosaccharides. Appl. Environ. Microbiol. 73, 3637–3644. 10.1128/AEM.02914-06 17434997 PMC1932670

[B3] Andersen S. J. Hennebel T. Gildemyn S. Coma M. Desloover J. Berton J. (2014). Electrolytic membrane extraction enables production of fine chemicals from biorefinery sidestreams. Environ. Sci. Technol. 48, 7135–7142. 10.1021/es500483w 24844669

[B4] Andrews J. H. Harris R. F. (1986). R- and K-selection in microbial ecology. Adv. Microb. Ecol. 9, 99–147.

[B5] Angenent L. T. Richter H. Buckel W. Spirito C. M. Steinbusch K. J. J. Plugge C. M. (2016). Chain elongation with reactor microbiomes: open-culture biotechnology to produce biochemicals. Environ. Sci. Technol. 50, 2796–2810. 10.1021/acs.est.5b04847 26854969

[B6] APHA (1992). Standard methods for the examination of water and wastewater. Washington DC, USA: 18th Editi. American Public Health Association.

[B7] Arancon R. A. D. Lin C. S. K. Chan K. M. Kwan T. H. Luque R. (2013). Advances on waste valorization: new horizons for a more sustainable society. Energy Sci. Eng. 1, 53–71. 10.1002/ese3.9

[B8] Bühlmann C. H. Mickan B. S. Tait S. Batstone D. J. Mercer G. D. Bahri P. A. (2022). Lactic acid from mixed food waste fermentation using an adapted inoculum: influence of pH and temperature regulation on yield and product spectrum. J. Clean. Prod. 373, 133716. 10.1016/j.jclepro.2022.133716

[B9] Candry P. Chadwick G. L. Caravajal-Arroyo J. M. Lacoere T. Winkler M. K. H. Ganigué R. (2023). Trophic interactions shape the spatial organization of medium-chain carboxylic acid producing granular biofilm communities. ISME J. 17, 2014–2022. 10.1038/s41396-023-01508-8 37715042 PMC10579388

[B10] Candry P. Ganigué R. (2021). Chain elongators, friends, and foes. Curr. Opin. Biotechnol. 67, 99–110. 10.1016/j.copbio.2021.01.005 33529974

[B11] Candry P. Radić L. Favere J. Carvajal-Arroyo J. M. Rabaey K. Ganigué R. (2020). Mildly acidic pH selects for chain elongation to caproic acid over alternative pathways during lactic acid fermentation. Water Res. 186, 116396. 10.1016/j.watres.2020.116396 32920334

[B12] Candry P. Van Daele T. Denis K. Amerlinck Y. Andersen S. J. Ganigué R. (2018). A novel high-throughput method for kinetic characterisation of anaerobic bioproduction strains, applied to Clostridium kluyveri. Sci. Rep. 8. 10.1038/s41598-018-27594-9 PMC6021416 29950677

[B13] Carvajal-Arroyo J. M. Candry P. Andersen S. J. Props R. Seviour T. Ganigué R. (2019). Granular fermentation enables high rate caproic acid production from solid-free thin stillage. Green Chem. 21, 1330–1339. 10.1039/c8gc03648a

[B14] Cavalcante W. de A. Leitão R. C. Gehring T. A. Angenent L. T. Santaella S. T. (2017). Anaerobic fermentation for n-caproic acid production: a review. Process Biochem. 54, 106–119. 10.1016/j.procbio.2016.12.024

[B15] Colombo B. Favini F. Scaglia B. Sciarria T. P. D’Imporzano G. Pognani M. (2017). Enhanced polyhydroxyalkanoate (PHA) production from the organic fraction of municipal solid waste by using mixed microbial culture. Biotechnol. Biofuels 10, 201. 10.1186/s13068-017-0888-8 28852422 PMC5567430

[B16] De Boer J. P. Teixeira De Mattos M. J. Neijssel O. M. (1990). D(-)Lactic acid production by suspended and aggregated continuous cultures of Bacillus laevolacticus. Appl. Microbiol. Biotechnol. 34, 149–153. 10.1007/bf00166771

[B17] De Groof V. Coma M. Arnot T. C. Leak D. J. Lanham A. B. (2020). Adjusting organic load as a strategy to direct single-stage food waste fermentation from anaerobic digestion to chain elongation. Processes 8. 10.3390/pr8111487

[B18] De Paz L. E. C. Bergenholtz G. Dahlén G. Svensäter G. (2007). Response to alkaline stress by root canal bacteria in biofilms. Int. Endod. J. 40, 344–355. 10.1111/j.1365-2591.2006.01226.x 17326786

[B19] Diender M. Stams A. J. M. Sousa D. Z. (2016). Production of medium-chain fatty acids and higher alcohols by a synthetic co-culture grown on carbon monoxide or syngas. Biotechnol. Biofuels 9, 82. 10.1186/s13068-016-0495-0 27042211 PMC4818930

[B20] Esquivel-Elizondo S. Bağcı C. Temovska M. Jeon B. S. Bessarab I. Williams R. B. H. (2021). The isolate caproiciproducens sp. 7D4C2 produces n-caproate at mildly acidic conditions from hexoses: genome and rBOX comparison with related strains and chain-elongating bacteria. Front. Microbiol. 11, 594524. 10.3389/fmicb.2020.594524 33584563 PMC7873966

[B21] Flaiz M. Baur T. Brahner S. Poehlein A. Daniel R. Bengelsdorf F. R. (2020). Caproicibacter fermentans gen. Nov., sp. nov., a new caproate-producing bacterium and emended description of the genus caproiciproducens. Int. J. Syst. Evol. Microbiol. 70, 4269–4279. 10.1099/ijsem.0.004283 32584751

[B22] Fong J. N. C. Yildiz F. H. (2015). Biofilm matrix proteins. Microbiol. Spectr. 3. 10.1128/microbiolspec.mb-0004-2014 PMC4480581 26104709

[B23] Fredborg M. Andersen K. R. Jørgensen E. Droce A. Olesen T. Jensen B. B. (2013). Real-time optical antimicrobial susceptibility testing. J. Clin. Microbiol. 51, 2047–2053. 10.1128/JCM.00440-13 23596243 PMC3697729

[B24] Ge S. Usack J. G. Spirito C. M. Angenent L. T. (2015). Long-term n-caproic acid production from yeast-fermentation beer in an anaerobic bioreactor with continuous product extraction. Environ. Sci. Technol. 49, 8012–8021. 10.1021/acs.est.5b00238 25941741

[B25] Goldin B. R. Gorbach S. L. Barakat S. Gualtieri L. Salminen S. (1992). Survival of lactobacillus species (strain GG) in human gastrointestinal tract. Dig. Dis. Sci. 37, 121–128. 10.1007/bf01308354 1728516

[B26] Greses S. Tomás-Pejó E. González-Fernández C. (2022). Food waste valorization into bioenergy and bioproducts through a cascade combination of bioprocesses using anaerobic open mixed cultures. J. Clean. Prod. 372, 133680. 10.1016/j.jclepro.2022.133680

[B27] Gu Y. Zhu X. Lin F. Shen C. Li Y. Ao L. (2021). Caproicibacterium amylolyticum gen. Nov., sp. nov., a novel member of the family oscillospiraceae isolated from pit clay used for making Chinese strong aroma-type liquor. Int. J. Syst. Evol. Microbiol. 71. 10.1099/ijsem.0.004789 33906707

[B28] Hall T. A. (1999). BioEdit: a user-friendly biological sequence alignment editor and analysis program for Windows 95/98/NT 1999. Nucleic Acids Symp. Ser. 41, 95 —98.

[B29] Hinshelwood C. N. (1946). The chemical kinetics of the bacterial cell. New York: Oxford University Press.

[B30] Hulshoff Pol L. W. De Castro Lopes S. I. Lettinga G. Lens P. N. L. (2004). Anaerobic sludge granulation. Water Res. 38, 1376–1389. 10.1016/j.watres.2003.12.002 15016515

[B31] Iragavarapu G. P. Imam S. S. Sarkar O. Mohan S. V. Chang Y. C. Reddy M. V. (2023). Bioprocessing of waste for renewable chemicals and fuels to promote bioeconomy. Energies 16, 3873. 10.3390/en16093873

[B32] Jia Y. Yang B. Ross P. Stanton C. Zhang H. Zhao J. (2020). Comparative genomics analysis of lactobacillus mucosae from different niches. Genes (Basel) 11, 95. 10.3390/genes11010095 31947593 PMC7016874

[B33] Kanehisa M. Furumichi M. Sato Y. Kawashima M. Ishiguro-Watanabe M. (2023). KEGG for taxonomy-based analysis of pathways and genomes. Nucleic Acids Res. 51, D587–D592. 10.1093/nar/gkac963 36300620 PMC9825424

[B34] Kang S. Kim H. Jeon B. S. Choi O. Sang B. I. (2022). Chain elongation process for caproate production using lactate as electron donor in Megasphaera hexanoica. Bioresour. Technol. 346, 126660. 10.1016/j.biortech.2021.126660 34974100

[B35] Kim B. C. Jeon B. S. Kim S. Kim H. Um Y. Sang B. I. (2015). Caproiciproducens galactitolivorans gen. Nov., sp. nov., a bacterium capable of producing caproic acid from galactitol, isolated from a wastewater treatment plant. Int. J. Syst. Evol. Microbiol. 65, 4902–4908. 10.1099/ijsem.0.000665 26474980

[B36] Kim D.-H. Lee M.-K. Hwang Y. Im W.-T. Yun Y.-M. Park C. (2016). Microbial granulation for lactic acid production. Biotechnol. Bioeng. 113, 101–111. 10.1002/bit.25540 25925200

[B37] Klappenbach J. A. Dunbar J. M. Schmidt T. M. (2000). rRNA operon copy number reflects ecological strategies of bacteria. Appl. Environ. Microbiol. 66, 1328–1333. 10.1128/aem.66.4.1328-1333.2000 10742207 PMC91988

[B38] Klindworth A. Pruesse E. Schweer T. Peplies J. Quast C. Horn M. (2013). Evaluation of general 16S ribosomal RNA gene PCR primers for classical and next-generation sequencing-based diversity studies. Nucleic Acids Res. 41, e1. 10.1093/nar/gks808 22933715 PMC3592464

[B39] Kozich J. J. Westcott S. L. Baxter N. T. Highlander S. K. Schloss P. D. (2013). Development of a dual-index sequencing strategy and curation pipeline for analyzing amplicon sequence data on the miseq illumina sequencing platform. Appl. Environ. Microbiol. 79, 5112–5120. 10.1128/AEM.01043-13 23793624 PMC3753973

[B40] Kreft J. U. Griffin B. M. González-Cabaleiro R. (2020). Evolutionary causes and consequences of metabolic division of labour: why anaerobes do and aerobes don’t. Curr. Opin. Biotechnol. 62, 80–87. 10.1016/j.copbio.2019.08.008 31654858

[B41] Kubota H. Senda S. Nomura N. Tokuda H. Uchiyama H. (2008). Biofilm Formation by lactic acid bacteria and resistance to environmental stress. J. Biosci. Bioeng. 106, 381–386. 10.1263/jbb.106.381 19000615

[B42] Lambrecht J. Cichocki N. Schattenberg F. Kleinsteuber S. Harms H. Müller S. (2019). Key sub-community dynamics of medium-chain carboxylate production. Microb. Cell Fact. 18, 92. 10.1186/s12934-019-1143-8 31138218 PMC6537167

[B43] Lawson C. E. Harcombe W. R. Hatzenpichler R. Lindemann S. R. Löffler F. E. O’Malley M. (2019). Common principles and best practices for engineering microbiomes. Nat. Rev. Microbiol. 17, 725–741. 10.1038/s41579-019-0255-9 31548653 PMC8323346

[B44] Lee J. H. Valeriano V. D. Shin Y. R. Chae J. P. Kim G. B. Ham J. S. (2012). Genome sequence of Lactobacillus mucosae LM1, isolated from piglet feces. J. Bacteriol. 194, 4766. 10.1128/JB.01011-12 22887668 PMC3415503

[B45] Liu C. Du Y. Zheng J. Qiao Z. Luo H. Zou W. (2022). Production of caproic acid by Rummeliibacillus suwonensis 3B-1 isolated from the pit mud of strong-flavor baijiu. J. Biotechnol. 358, 33–40. 10.1016/j.jbiotec.2022.08.017 36049550

[B46] Liu S. (2017). Bioprocess engineering: kinetics, sustainability, and reactor design. 2nd ed. Elsevier.

[B47] Madden T. L. Tatusov R. L. Zhang J. (1996). Applications of network BLAST server. Meth Enzymol. 266, 131–141. 10.1016/s0076-6879(96)66011-x 8743682

[B48] Mariën Q. Candry P. Hendriks E. Carvajal-Arroyo J. M. Ganigué R. (2022a). Substrate loading and nutrient composition steer caproic acid production and biofilm aggregation in high-rate granular reactors. J. Environ. Chem. Eng. 10, 107727. 10.1016/j.jece.2022.107727

[B49] Mariën Q. Ulčar B. Verleyen J. Vanthuyne B. Ganigué R. (2022b). High-rate conversion of lactic acid-rich streams to caproic acid in a fermentative granular system. Bioresour. Technol. 355, 127250. 10.1016/j.biortech.2022.127250 35562021

[B50] Moscoviz R. Trably E. Bernet N. Carrère H. (2018). The environmental biorefinery: state-of-the-art on the production of hydrogen and value-added biomolecules in mixed-culture fermentation. Green Chem. 20, 3159–3179. 10.1039/c8gc00572a

[B51] Nwodo U. U. Green E. Okoh A. I. (2012). Bacterial exopolysaccharides: functionality and prospects. Int. J. Mol. Sci. 13, 14002–14015. 10.3390/ijms131114002 23203046 PMC3509562

[B52] Nzeteu C. O. Trego A. C. Abram F. O’Flaherty V. (2018). Reproducible, high-yielding, biological caproate production from food waste using a single-phase anaerobic reactor system. Biotechnol. Biofuels 11, 108. 10.1186/s13068-018-1101-4 29651303 PMC5894149

[B53] Palmqvist E. Agerdal H.-H. (2000). Fermentation of lignocellulosic hydrolysates. II: inhibitors and mechanisms of inhibition. Bioresour. Technol. 74, 25–33. 10.1016/s0960-8524(99)00161-3

[B54] Pantaléon V. Bouttier S. Soavelomandroso A. P. Janoir C. Candela T. (2014). Biofilms of Clostridium species. Anaerobe 30, 193–198. 10.1016/j.anaerobe.2014.09.010 25242197

[B55] Park J. H. Kim D. H. Kim S. H. Yoon J. J. Park H. D. (2018). Effect of substrate concentration on the competition between Clostridium and Lactobacillus during biohydrogen production. Int. J. Hydrogen Energy 43, 11460–11469. 10.1016/j.ijhydene.2017.08.150

[B56] Pontius K. (2019). Monitoring of bioprocesses. Opportunities and challenges: opportunities and challenges. Kongens Lyngby: Technical University of Denmark. [dissertation].

[B57] Posit team (2023). RStudio. Boston, MA: Integrated Development Environment for R. Posit Software, PBC. URL: http://www.posit.co/.

[B58] R Core Team (2023). R: a language and environment for statistical computing. Vienna, Austria: R Foundation for Statistical Computing. https://www.R-project.org/.

[B59] Regueira A. Bevilacqua R. Mauricio-Iglesias M. Carballa M. Lema J. M. (2021). Kinetic and stoichiometric model for the computer-aided design of protein fermentation into volatile fatty acids. Chem. Eng. J. 406, 126835. 10.1016/j.cej.2020.126835

[B60] Rombouts J. L. Kranendonk E. M. M. Regueira A. Weissbrodt D. G. Kleerebezem R. van Loosdrecht M. C. M. (2020). Selecting for lactic acid producing and utilising bacteria in anaerobic enrichment cultures. Biotechnol. Bioeng. 117, 1281–1293. 10.1002/bit.27301 32034763 PMC7187302

[B61] Roos S. Karner F. Axelsson L. Jonsson H. (2000). Lactobacillus mucosae sp. nov., a new species with *in vitro* mucus-binding activity isolated from pig intestine. Int. J. Syst. Evol. Microbiol. 50, 251–258. 10.1099/00207713-50-1-251 10826811

[B62] Royce L. A. Liu P. Stebbins M. J. Hanson B. C. Jarboe L. R. (2013). The damaging effects of short chain fatty acids on *Escherichia coli* membranes. Appl. Microbiol. Biotechnol. 97, 8317–8327. 10.1007/s00253-013-5113-5 23912117 PMC3757260

[B84] Stadtman E. R. Barker H. A. (1949). Fatty acid synthesis by enzyme preparations of Clostridium kluyveri. I. Preparation of cell free extracts that catalyze the conversion of ethanol and acetate to butyrate and caproate. J. Biol. Chem. 180, 1085–1093. 18139204

[B63] Scarborough M. J. Lawson C. E. Hamilton J. J. Donohue T. J. Noguera D. R. (2018a). Metatranscriptomic and thermodynamic insights into medium-chain fatty acid production using an anaerobic microbiome. mSystems 3, 002211–e318. 10.1128/msystems.00221-18 PMC6247018 30505946

[B64] Scarborough M. J. Lynch G. Dickson M. McGee M. Donohue T. J. Noguera D. R. (2018b). Increasing the economic value of lignocellulosic stillage through medium-chain fatty acid production. Biotechnol. Biofuels 11, 200. 10.1186/s13068-018-1193-x 30034526 PMC6052542

[B65] Schloss P. D. Westcott S. L. Ryabin T. Hall J. R. Hartmann M. Hollister E. B. (2009). Introducing mothur: open-source, platform-independent, community-supported software for describing and comparing microbial communities. Appl. Environ. Microbiol. 75, 7537–7541. 10.1128/AEM.01541-09 19801464 PMC2786419

[B66] Spector M. P. (2009). Metabolism, central (intermediary). Encycl. Microbiol., 242–264. 10.1016/b978-012373944-5.00078-x

[B67] Tamis J. Joosse B. M. Van Loosdrecht M. C. M. Kleerebezem R. (2015). High-rate volatile fatty acid (VFA) production by a granular sludge process at low pH. Biotechnol. Bioeng. 112, 2248–2255. 10.1002/bit.25640 25950759

[B68] Tamura K. Stecher G. Kumar S. (2021). MEGA11: molecular evolutionary genetics analysis version 11. Mol. Biol. Evol. 38, 3022–3027. 10.1093/molbev/msab120 33892491 PMC8233496

[B69] Teusink B. Molenaar D. (2017). Systems biology of lactic acid bacteria: for food and thought. Curr. Opin. Syst. Biol. 6, 7–13. 10.1016/j.coisb.2017.07.005 32954057 PMC7489361

[B70] Thauer R. K. Jungermann K. Henninger H. Wenning J. Decker K. (1968). The energy metabolism of Clostridium kluyveri. Eur. J. Biochem. 4, 173–180. 10.1111/j.1432-1033.1968.tb00189.x 5655494

[B71] Trunk T. Khalil H. S. Leo J. C. (2018). Bacterial autoaggregation. AIMS Microbiol. 4, 140–164. 10.3934/microbiol.2018.1.140 31294207 PMC6605025

[B72] Vilchez-Vargas R. Geffers R. Suárez-Diez M. Conte I. Waliczek A. Kaser V. S. (2013). Analysis of the microbial gene landscape and transcriptome for aromatic pollutants and alkane degradation using a novel internally calibrated microarray system. Environ. Microbiol. 15, 1016–1039. 10.1111/j.1462-2920.2012.02752.x 22515215

[B73] Wallis J. K. Krömker V. Paduch J. H. (2018). Biofilm formation and adhesion to bovine udder epithelium of potentially probiotic lactic acid bacteria. AIMS Microbiol. 4, 209–224. 10.3934/MICROBIOL.2018.2.209 31294211 PMC6604931

[B74] Wang H. Gu Y. Zhao D. Qiao Z. Zheng J. Gao J. (2022a). Caproicibacterium lactatifermentans sp. nov., isolated from pit clay used for the production of Chinese strong aroma-type liquor. Int. J. Syst. Evol. Microbiol. 72. 10.1099/ijsem.0.005206 35085065

[B75] Wang H. Gu Y. Zhou W. Zhao D. Qiao Z. Zheng J. (2021a). Adaptability of a caproate-producing bacterium contributes to its dominance in an anaerobic fermentation system. Appl. Environ. Microbiol. 87, e0120321–21. 10.1128/AEM.01203-21 34378978 PMC8478455

[B76] Wang H. Zhou W. Gao J. Ren C. Xu Y. (2022b). Revealing the characteristics of glucose- and lactate-based chain elongation for caproate production by Caproicibacterium lactatifermentans through transcriptomic, bioenergetic, and regulatory analyses. mSystems 7, e0053422. 10.1128/msystems.00534-22 36073803 PMC9600882

[B77] Wang J. Huang J. Jiang S. Zhang J. Zhang Q. Ning Y. (2021b). Parametric optimization and kinetic study of l-lactic acid production by homologous batch fermentation of Lactobacillus pentosus cells. Biotechnol. Appl. Biochem. 68, 809–822. 10.1002/bab.1994 32738151

[B78] Wang Q. Garrity G. M. Tiedje J. M. Cole J. R. (2007). Naïve Bayesian classifier for rapid assignment of rRNA sequences into the new bacterial taxonomy. Appl. Environ. Microbiol. 73, 5261–5267. 10.1128/AEM.00062-07 17586664 PMC1950982

[B79] Yáñez R. Marques S. Gírio F. M. Roseiro J. C. (2008). The effect of acid stress on lactate production and growth kinetics in Lactobacillus rhamnosus cultures. Process Biochem. 43, 356–361. 10.1016/j.procbio.2007.12.014

[B80] Yin Q. Sun Y. Li B. Feng Z. Wu G. (2022). The r/K selection theory and its application in biological wastewater treatment processes. Sci. Total Environ. 824, 153836. 10.1016/j.scitotenv.2022.153836 35176382

[B81] Youssef C. B. Goma G. Olmos-Dichara A. (2005). Kinetic modelling of Lactobacillus casei ssp. rhamnosus growth and lactic acid production in batch cultures under various medium conditions. Biotechnol. Lett. 27, 1785–1789. 10.1007/s10529-005-3557-0 16314971

[B82] Zheng J. Wittouck S. Salvetti E. Franz C. M. A. P. Harris H. M. B. Mattarelli P. (2020). A taxonomic note on the genus Lactobacillus: description of 23 novel genera, emended description of the genus Lactobacillus beijerinck 1901, and union of Lactobacillaceae and Leuconostocaceae. Int. J. Syst. Evol. Microbiol. 70, 2782–2858. 10.1099/ijsem.0.004107 32293557

[B83] Zwietering M. H. Jongenburger I. Rombouts F. M. Van ’T Riet K. (1990). Modeling of the bacterial growth curve. Appl. Environ. Microbiol. 56, 1875–1881. 10.1128/aem.56.6.1875-1881.1990 16348228 PMC184525

