# Supplementary material for: Why do lactic acid bacteria thrive in chain elongation microbiomes?
Source: Front Bioeng Biotechnol. 2024 Jan 11;11:1291007. doi: 10.3389/fbioe.2023.1291007 (PMC10809155; doi:10.3389/fbioe.2023.1291007)
Supplement: Supplementary file 2 [file DataSheet1.DOCX]

Supplementary Material

Why do lactic acid bacteria thrive in chain elongation microbiomes?

Barbara Ulčar, Alberte Regueira, Maja Podojsteršek, Nico Boon, Ramon Ganigué^*^

*** Correspondence:** Ramon Ganigué: Ramon.Ganigue@UGent.Be

# Supplementary Materials and Methods

## BGM medium preparation

The developed minimal medium called Basic Glucose Medium (BGM) enabled precise control over the dosed carbon source and was a combination of the reactor medium (SI Table 1) and of a widely used PYG modified medium (DSMZ medium 104). The medium contained, per liter: 4.0 g tryptone, 2 g K_2_HPO_4_, 0.001 g resazurin and 40 ml PYG (DSMZ medium 104) salt solution (without K_2_HPO_4_). PYG salt solution composed of, per liter: 0.25 g CaCl_2_.2H_2_O, 0.5 g MgSO_4_.7H2O, 1.0 g KH_2_PO4, 10 g NaHCO_3_, 2.0 g NaCl. Additionally, 0.1 M organic acid was used to maintain the pH at 5.5; either 11.81 g/L of succinic acid (BGM-SUC) or 8.20 g/L of sodium acetate (BGM-AC) was used. All the reagents were mixed with distillated water, boiled and distributed with constant sparging into serum flasks (95 % of the final volume was added). The headspace was replaced to N_2_ atmosphere and the serum flasks were autoclaved.

Prior to the inoculation, the rest of the medium components were added anaerobically and sterile to the basal medium. These additions were prepared in 20-times concentrate, dissolved in the basal medium, anaerobically (in the N_2_ serum bottle) filter-sterilized (0.22 μm filter) and added to the serum bottles (contributing final 5 % of the medium volume). The non-concentrated additions contained per liter: 2.2 g glucose.H_2_O, 0.2 g yeast extract, 0.278 g Na-acetate, 0.5 g Cysteine-HCl.H_2_O, 0.2 ml vitamin K_1_, 1 mL of trace element solution SL-10, 1 mL of selenite-tungstate solution and 1 mL of 7-vitamin solution. The SL-10 trace element solution consisted of, per liter: 10 mL 7.7 M HCl, 1.5 g FeCl_2_.4H_2_O, 0.07 g ZnCl_2_, 0.15 g MnCl_2_.4H_2_O, 0.006 g H_3_BO_3_, 0.19 g CoCl_2_.6H_2_O, 0.002 g CuCl_2_.2H_2_O, 0.024 g NiCl_2_.6H_2_O and 0.036 g Na_2_MoO_4_.4H_2_O. The selenite-tungstate solution consisted of, per liter: 0.5 g NaOH, 0.003 g Na_2_SeO_3_.5H_2_O and 0.004 g Na_2_WO_4_.2H_2_O. The 7-vitamin solution consisted of, per liter: 0.1 g vitamin B_12_, 0.08 g p-aminobenzoic acid, 0.02 g D(+)-Biotin, 0.2 g nicotinic acid, 0.1 g Ca-pantothenate, 0.3 g pyridoxine hydrochloride, 0.2 g thiamine-HCl.2H_2_O. Vitamin K_1_ was prepared by dissolving 0.1 ml of vitamin K_1_ in 20 ml of 95 % ethanol, filter-sterilized (0.22 µm filter) and stored refrigerated in a brown bottle. Titanium (III) citrate (Ti-citrate) was used as a reducing agent and was added immediately prior the inoculation, 0.05 ml/bottle was used. It was prepared by mixing 10 g of Na-citrate and 5 mL of TiCl3 (15 % solution) with 40 ml of distilled H2O, stabilizing the pH at 7 with NaOH, anaerobically (in a N2 serum bottle) filter-sterilizing (0.22 μm filter) and storing the solution at 4 °C in the dark.

# Supplementary Figures and Tables

## Supplementary Tables

Supplementary Table 1: Medium used in the reactor operation.

| **Compound** | **Concentration (g/l)** |
| --- | --- |
| MgCl_2_.6H_2_O | 2.18 |
| CaCl_2_.2H_2_O | 0.73 |
| NaH_2_PO_4_.2H_2_O | 1.64 |
| Na_2_SO_4_ | 0.11 |
| KCl | 6.7 |
| NH_4_Cl | 1.91 |
| yeast extract | 1 |
| Tryptone | 4 |
| Glucose.1H20 | 20 |
| SL-10 trace elements | 10 mL |
| Se-W-solution | 10 mL |
| 7-vitamin solution (10x) | 0.1 mL |

Table 2: Overview of tested medium iterations. Different parameters were tested in each phase, the final values of choice are labelled in bold. The average values for the biomass (OD_600_) and pH changes are shown (Phase I: n=2, Phase II: n=4, Phase III: n=1).

| Phase | Organism | SL-10 (mL/L) | SE-W (mL/L) | 7-vitamins (mL/L) | Glucose concentration (g/L) | Yeast extract (g/L) | Ratio glucose / yeast extract | Buffer | Change in OD_600_ | Change in pH |
| --- | --- | --- | --- | --- | --- | --- | --- | --- | --- | --- |
| I | *L. mucosae* G03 | 1 | 1 | 1 | 5 | 1 | 5 | Acetic acid | 0.66 | NA |
|  | *L. mucosae* G03 | **1** | **1** | **1** | 5 | 0.5 | **10** | Acetic acid | 0.65 | NA |
|  | *L. mucosae* G03 | 0 | 0 | 0 | 5 | 1 | 5 | Acetic acid | 0.03 | NA |
|  | *L. mucosae* G03 | 0 | 0 | 0 | 5 | 0.5 | 10 | Acetic acid | 0.06 | NA |
| II | *L. mucosae* G03 | 1 | 1 | 1 | 5 | 0.5 | 10 | Acetic acid | 0.21 | -0.6 |
|  | *L. mucosae* G03 | 1 | 1 | 1 | **2** | **0.2** | 10 | Acetic acid | 0.11 | -0.4 |
| III | *C. lactatifermentans* | 1 | 1 | 1 | 2 | 0.2 | 10 | Acetic acid | 0.61 | -0.3 |
|  | *C. lactatifermentans* | 1 | 1 | 1 | 2 | 0.2 | 10 | **Succinic acid** | 0.49 | -0.1 |
|  | *C. lactatifermentans* | 1 | 1 | 1 | 2 | 0.2 | 10 | Malonic acid | 0.28 | 0 |
|  | *C. lactatifermentans* | 1 | 1 | 1 | 2 | 0.2 | 10 | Citric acid | -0.08 | 0.1 |

Table 3: The pH value and gas production in the experiments for determination of growth rates and biomass yields for *L. mucosae* G03 and *C. lactatifermentans* (n=3).

|  | **Initial pH** | **Final pH** | **∆ Pressure (kPa)** | **CO_2_ (%)** | **H_2_ (%)** | **N_2_ (%)** |
| --- | --- | --- | --- | --- | --- | --- |
| *L. mucosae* G03 | 5.53 ± 0.01 | 5.46 ± 0.02 | 17.8 ± 1.0 | 10.0 ± 0.2 | 0 | 90.5 ± 0.7 |
| *C. lactatifermentans* | 5.53 ± 0.01 | 5.53 ± 0.02 | 48.4 ± 2.4 | 16.2 ± 0.2 | 14.0 ± 0.7 | 70.9 ± 0.7 |

## Supplementary Figures


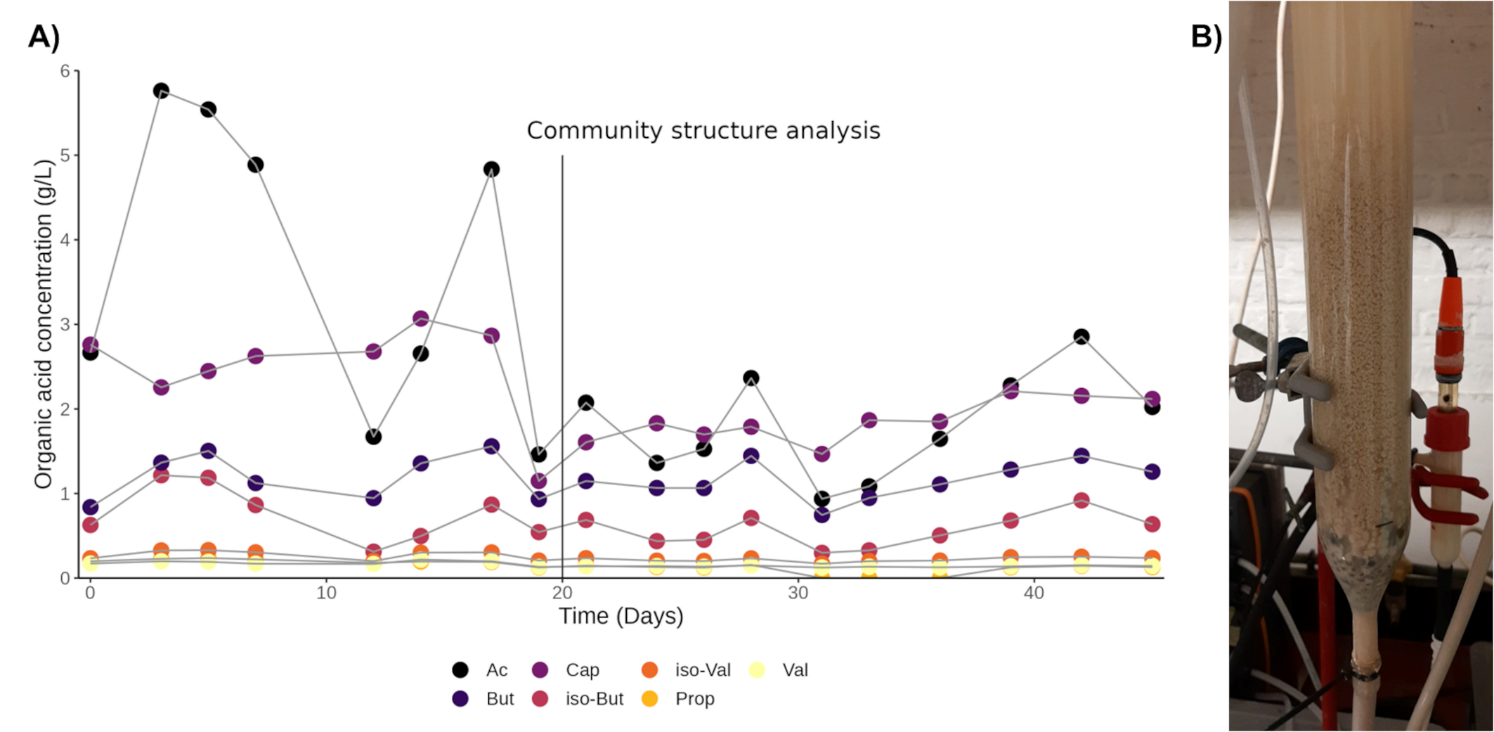


Supplementary Figure 1: A) Product spectrum during the reactor operation. The line indicates the time point when community structure of the granular biomass was analyzed. (Ac – acetate, Prop – propionate, But – butyrate, iso-But – iso-butyrate, Val – valerate, Cap – caproate). B) Photo of the reactor with granulated biomass on the day 32.


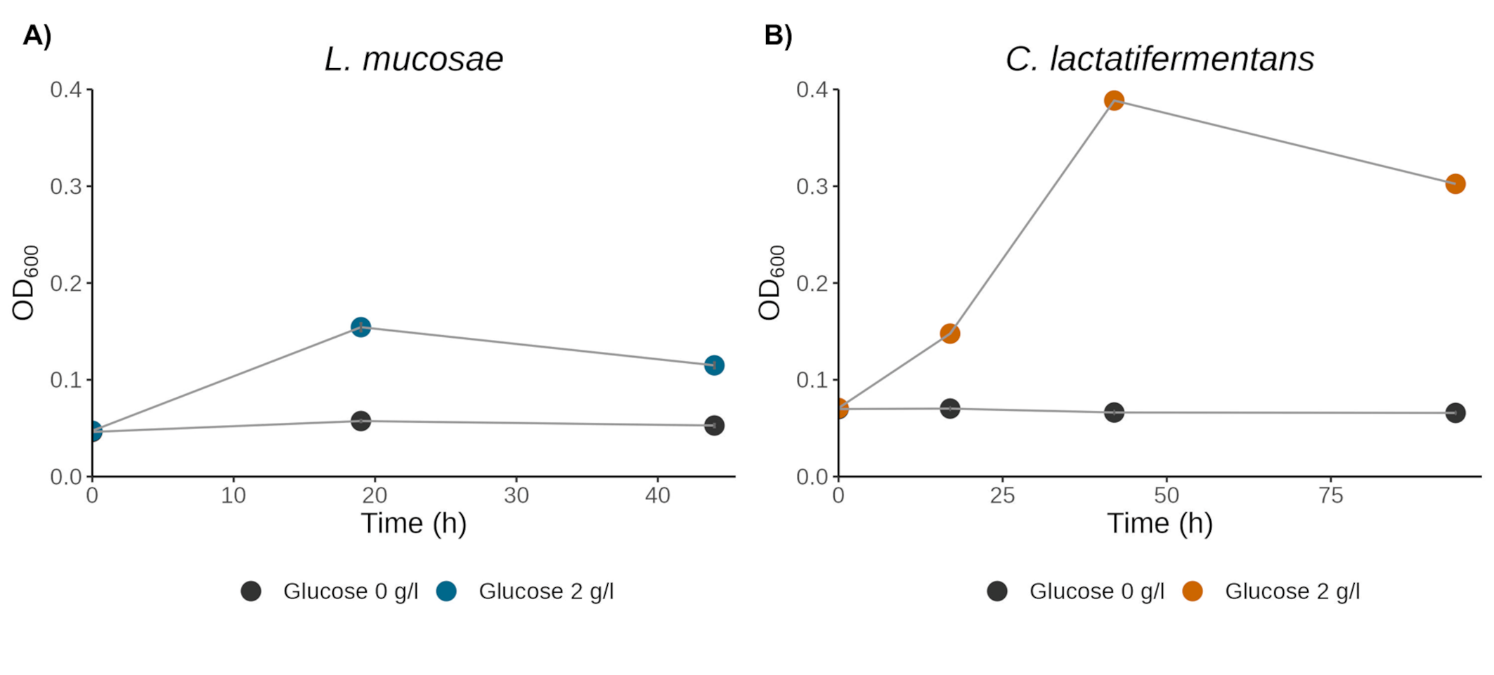


Supplementary Figure 2: Growth of the cultures in the absence of glucose (0 g/L). Same inoculum was transferred to the complete medium (with 2 g/L of glucose) to ensure that the cultures were active. Average values are shown and the error bars represent standard deviation (n=3).


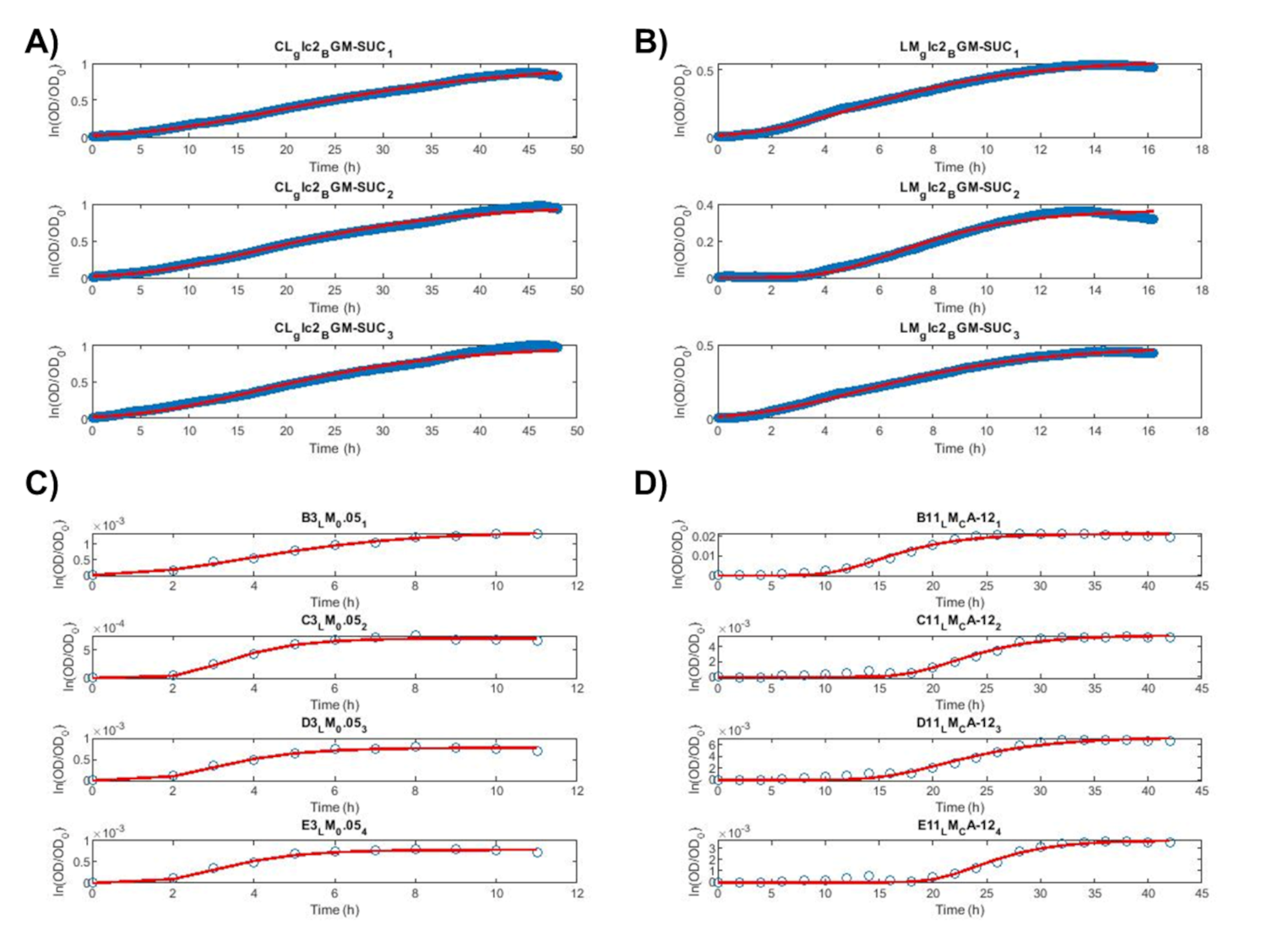


Supplementary Figure 3: Fit of the Gompertz equation to the growth curves. A) Growth of *C.* lactatifermentans in BGM-SUC medium with 2 g/L glucose, monitored with the CGQuant device (n=3). B) Growth of *L. mucosae* in BGM-SUC medium with 2 g/L glucose monitored with the CGQuant device (n=3). C) Growth of *L. mucosae* in BGM-SUC medium with 0.05 g/L glucose monitored with the oCelloScope device (n=4). D) Growth of *L. mucosae* in BGM-SUC medium with 2 g/L glucose and 12 g/L caproic acid monitored with the oCelloScope device (n=4).


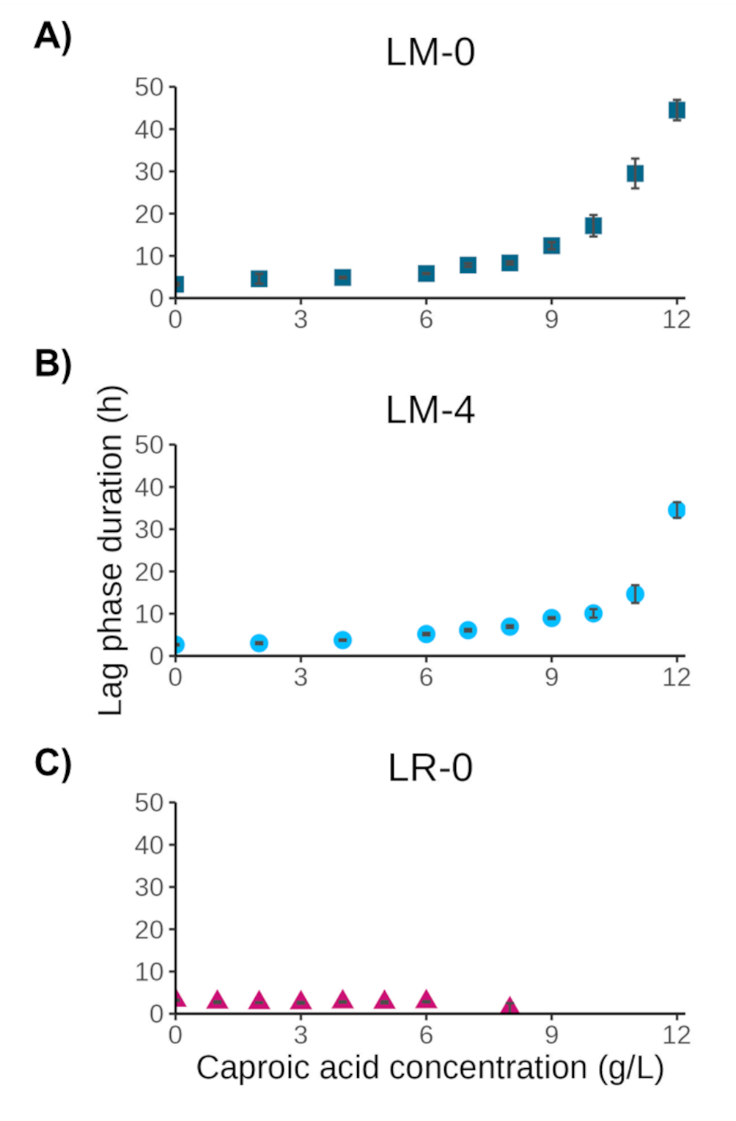


Supplementary Figure 4: Impact of caproic acid on the duration of lag phase for three different cultures. Average values are shown with error bars representing the standard deviations. A) isolate *L. mucosae* G03 (LM-0) (n = 3). B) isolate *L. mucosae* G03 adjusted to 4 g/L of CA (LM-4) (n=4). C) type strain of *L. rhamnosus* (LR-0) (n=4).


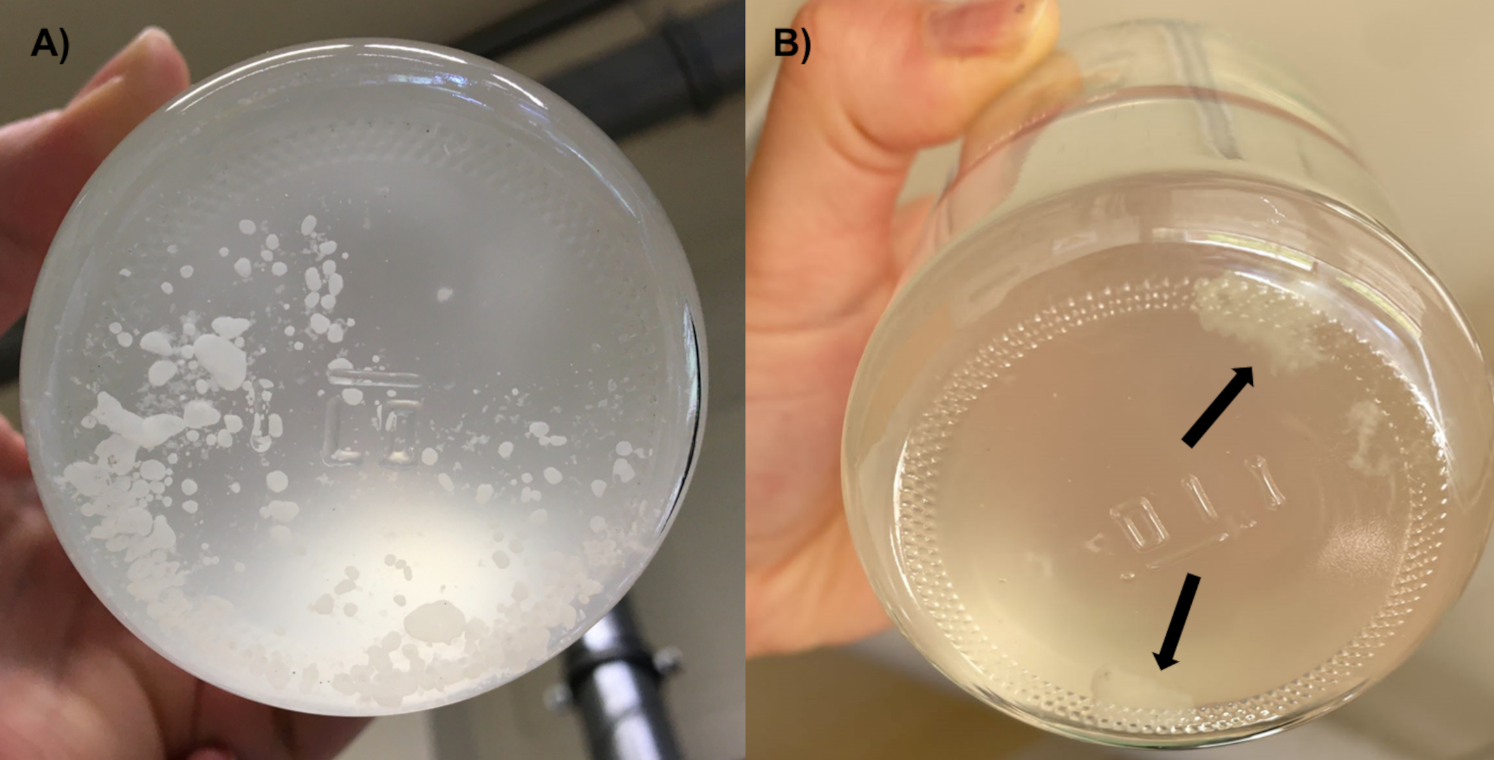


Supplementary Figure 5: Photo of the biomass aggregation. A) *L. mucosae* RG03 (BGM-SUC medium, 5 g/L glucose, shaking). B) *C. lactatifermentans* culture (BGM-AC medium, 2 g/L glucose, initial pH 6.3, shaking).
